# Effects of Quercetin and Resveratrol on *in vitro* Properties Related to the Functionality of Potentially Probiotic *Lactobacillus* Strains

**DOI:** 10.3389/fmicb.2019.02229

**Published:** 2019-09-24

**Authors:** Aldeir Sabino dos Santos, Thatyane Mariano Rodrigues de Albuquerque, José Luiz de Brito Alves, Evandro Leite de Souza

**Affiliations:** Department of Nutrition, Health Sciences Center, Federal University of Paraíba, João Pessoa, Brazil

**Keywords:** polyphenols, stilbenes, flavonoids, probiotics, physiological properties, modulatory effects

## Abstract

The ability of probiotics to exert benefits on host has been associated with different physiological functionalities in these microorganisms, namely cell surface hydrophobicity, autoaggregation, coaggregation with pathogens, antagonistic activity against pathogens and ability to survive the exposure to gastrointestinal conditions. This study assessed the effects of different concentrations of quercetin (QUE) and resveratrol (RES) on the ability of six potentially probiotic *Lactobacillus* strains to tolerate different pH values and bile salt concentrations, to autoaggregate, coaggregate with and antagonize pathogens and survive the exposure to simulated gastrointestinal conditions. QUE and RES presented low inhibitory effects on all tested *Lactobacillus* strains, with minimum inhibitory concentration (MIC) ranging from 512 to >1024 μg/mL. In most cases, QUE and RES at all tested concentrations (i.e., MIC, 1/2 MIC, and 1/4 MIC) did not affect the tolerance of the *Lactobacillus* strains to acidic pH and bile salts. QUE increased cell surface hydrophobicity of most of the tested *Lactobacillus* strains and increases or decreases in cell surface hydrophobicity varied in the presence of different RES concentrations among some strains. QUE and RES increased the ability of tested *Lactobacillus* strains to autoaggregate and coaggregate with pathogens. QUE and RES did not negatively affect the antagonistic activity of the tested *Lactobacillus* strains against pathogens and did not decrease their survival rates when exposed to *in vitro* gastrointestinal conditions. In a few cases, the ability of some tested *Lactobacillus* strains to antagonize pathogens, as well as to survive specific steps of the *in vitro* digestion was increased by QUE and RES. QUE exerted overall better protective effects on the measured *in vitro* properties of tested *Lactobacillus* strains than RES, and *L. fermentum* and *L. plantarum* strains presented better responses when treated with QUE or RES. These results showed that probiotic *Lactobacillus* strains could present low susceptibility to QUE and RES. Combined use of QUE and RES with probiotic *Lactobacillus* could improve their functionalities on the host; however, the concentration of these polyphenols should be carefully selected to achieve the desirable effects and vary according to the selected probiotic strain.

## Introduction

Polyphenols are widely distributed in fruits, vegetables, herbs, seeds, cereals, honey and beverages (Hossen et al., 2017). Among the polyphenols present in foods, quercetin (QUE) and resveratrol (RES) have received increased interest because of their antioxidant and anti-inflammatory properties and evidence associating their intake with the prevention of non-communicable diseases, such as diabetes, obesity and cardiovascular disorders (Dolinsky et al., 2013; Gelen et al., 2017).

Quercetin is one of the major representatives of the class of flavonoids, being naturally found in apples and red wine. RES is the main representative of the class of stilbenes, being naturally present in bark and seeds of grapes and wine (Dolinsky et al., 2013; de Souza E.L. et al., 2019). Available literature reports that QUE and RES even at high concentrations (e.g., 1000–1500 mg/day) are well tolerated by mammals and exert no adverse health effects (Harwood et al., 2007; Sergides et al., 2016), increasing their potential clinical and food applications.

Polyphenols may exert health benefits at a local level when they act directly during passage through the gastrointestinal tract or at a systemic level after their absorption (Gelen et al., 2017). The digestion and absorption of dietary polyphenols are limited, being estimated that only 5–10% of total ingested polyphenols can be absorbed in the small intestine (Cueva et al., 2017). Consequently, high amounts of dietary polyphenols remain available for interaction with and/or use by microorganisms forming the gut microbiota (Moreno-Indias et al., 2016; de Souza E.L. et al., 2019).

*Lactobacillus* species comprise one of the main microbial groups of the gut microbiota of mammals (Ventura et al., 2009; David et al., 2014), in addition to be extensively studied for the selection of probiotic strains (de Albuquerque et al., 2017) and recently for their ability to metabolize polyphenols (Llano et al., 2017; Mueller et al., 2018). The potential ability of probiotics to exert health benefits on the host has been commonly associated with specific physiological functionalities (e.g., cell surface hydrophobicity, autoaggregation, coaggregation with pathogens and antagonistic activity against pathogens) and ability to survive during exposure to gastrointestinal conditions (Abushelaibi et al., 2017; de Albuquerque et al., 2017; de Souza B.M.S. et al., 2019). Consequently, possible negative impacts on these physiological functionalities in probiotics as result of their interaction with compounds coexisting in the same environment could be potential influential factors to reach the desired beneficial effects on the host (de Souza E.L. et al., 2019).

Although some researchers have proposed the combined use of dietary polyphenols and probiotic *Lactobacillus* as an advantageous strategy to enhance mutually their potential health-promoting properties (Lacey et al., 2014; Georgakouli et al., 2016), studies evaluating the influence of RES and QUE on the growth and probiotic-related *in vitro* properties of these bacteria remain scarce. Only a few studies have evaluated the influence of polyphenol-rich foods/beverages or other individual polyphenols on the growth and/or some probiotic-related *in vitro* properties of lactic acid bacteria, including *Lactobacillus* species (Llano et al., 2017; Chan et al., 2018; Pacheco-Ordaz et al., 2018).

This study first evaluated the susceptibility of six potentially probiotic *Lactobacillus* strains to QUE and RES. Second, the *in vitro* effects of different amounts of QUE and RES on the ability of these *Lactobacillus* strains to tolerate different pH values and bile salt concentrations, as well as on their ability to autoaggregate, coaggregate with and antagonize pathogens were evaluated. Finally, the effects of QUE and RES on the survival of the tested *Lactobacillus* strains when exposed to simulated gastrointestinal conditions were assessed.

## Materials and Methods

### Test Strains and Inoculum Preparation

Six *Lactobacillus* strains, namely *L. plantarum* 49, *L. plantarum* 53, *L. paracasei* 106, *L. paracasei* 108, *L. fermentum* 263, and *L. fermentum* 296, previously characterized as potentially probiotic in a series of safety (antibiotic resistance, mucinolytic, and hemolytic activity) and physiological functionality *in vitro* tests (bile salt deconjugation, cell surface hydrophobicity, autoaggregation, coaggregation with pathogens, acidic pH tolerance, bile salts tolerance, and survival to simulated gastrointestinal conditions) were used in this study (Garcia et al., 2016; de Albuquerque et al., 2017). These *Lactobacillus* species have been extensively reported as being part of human gut microbiota (Ventura et al., 2009; David et al., 2014). The tested *Lactobacillus* strains were previously identified using 16S rRNA gene sequence analysis (Garcia et al., 2016). Stocks were stored at −20°C in de Mann, Rogosa and Sharpe (MRS) broth (HiMedia, Mumbai, India) with glycerol (Sigma-Aldrich, St. Louis, MO, United States; 20 mL/100 mL). Working cultures were maintained aerobically on MRS agar (HiMedia, Mumbai, India) at 4 ± 0.5°C and transferred to new media monthly. Prior to use, each strain was cultivated anaerobically (using the AnaeroGen Anaerobic System, Oxoid, Hampshire, United Kingdom) in MRS broth at 37°C for 20–24 h to reach the stationary growth phase. These cultures were harvested through centrifugation (4500 *g* × 15 min, 4°C), washed twice and resuspended in sterile saline solution (0.85 g/100 mL) to obtain cell suspensions with an optical density reading at 625 nm (OD_625_) of 0.5. This suspension provided viable counts in the range of 7–8 log CFU/mL for each strain when enumerated on MRS agar (HiMedia, Mumbai, India). Each strain was tested separately as a single inoculum.

Strains of *Listeria monocytogenes* (INCQS 00266, originally ATCC 7644) and *Escherichia coli* (INCQS 00219, originally ATCC 8739) used in assays of antagonistic activity and coaggregation were obtained from the National Institute for Quality Control in Health (Oswaldo Cruz Foundation, Rio de Janeiro, Brazil). Stocks were stored in brain heart infusion (BHI) broth (HiMedia, Mumbai, India) with glycerol (Sigma-Aldrich, St. Louis, MO, United States; 20 mL/100 mL) at −20°C. Prior to use, each strain was aerobically grown in BHI broth at 37°C for 20–24 h to reach the stationary growth phase, harvested through centrifugation (4500 *g* × 15 min, and 4°C), washed twice and resuspended in sterile saline solution to obtain cell suspensions with an OD_625_ of 0.1. This suspension provided viable counts in the range of 7–8 log CFU/mL when enumerated on BHI agar (HiMedia, Mumbai, India). Each *L. monocytogenes* and *E. coli* strain was tested separately as a single inoculum.

### QUE and RES

Quercetin and resveratrol were obtained from Sigma-Aldrich (purity ≥ 95%; St. Louis, United States). The solutions of QUE and RES were prepared in MRS broth with dimethyl sulfoxide (DMSO, 10%, v/v) immediately before use in assays in an amount sufficient to provide initial QUE and RES concentrations of 2048 and 1400 μg/mL. QUE and RES were tested separately in all assays.

### Determination of the Minimum Inhibitory Concentration (MIC) of QUE and RES

The minimum inhibitory concentration values of QUE and RES on the tested *Lactobacillus* strains were determined using a microdilution in broth procedure (CLSI, 2012), with modification regarding the cultivation media. Initially, 100 μL-aliquots of the solutions with the different tested concentrations of QUE or RES were dispensed into wells of a 96-well microplate and each initial concentration was then serially diluted in MRS broth to provide at least eight different final concentrations. Subsequently, 100 μL of a suspension (7–8 log CFU/mL) of the test *Lactobacillus* strain was added to each well. The final tested concentrations of QUE and RES were in the range of 87.5–1024 μg/mL. The microplate with lid was anaerobically incubated (using the AnaeroGen Anaerobic System, Oxoid, Hampshire, United Kingdom) at 30°C for 24 h. Each microplate included a set of positive and negative controls. MIC was considered the lowest concentration of QUE and RES capable of causing visual growth inhibition of the target *Lactobacillus* strain.

### Assessment of the Effects of QUE and RES on Probiotic-Related *in vitro* Properties

#### Effects on Tolerance to Different pH Values and Bile Salt Concentrations

The tolerance to different pH values and bile salt concentrations was assessed by inoculating 1 mL-aliquots of the inoculum suspension of the tested *Lactobacillus* strain (grown anaerobically in MRS broth, 20–24 h, 37°C, using the AnaeroGen Anaerobic System, Oxoid, Hampshire, United Kingdom) in 10 mL PBS (50 mM K_2_HPO_4_; final viable counts in the range of 6–7 log CFU/mL) with different concentrations (MIC, 1/2 MIC or 1/4 MIC) of QUE or RES with pH adjusted to 2, 3, or 5 using 1 M HCl or supplemented with 0.15, 0.3 or 1% (w/v) bile salts (Sigma-Aldrich, St. Louis, MO, United States). The mixtures were incubated aerobically at 37°C under stirring (150 rpm). At different incubation time intervals (1–3 h), a 1 mL-aliquot was removed from each mixture, serially diluted in sterile peptone (0.15 g/100 mL) water and spread plated on MRS agar for enumeration of viable cells. After an incubation period of 48 h at 37°C under anaerobiosis (using the Anaerobic System AnaeroGen, Oxoid Hampshire, United Kingdom), the viable cells were enumerated and the results were expressed as the log CFU/mL. For controls, *Lactobacillus* strains were cultivated in PBS with pH 7.2 adjusted using 1 M NaOH, as well as in MRS without bile salts, QUE and RES (Jacobsen et al., 1999; Monteagudo-Mera et al., 2012). To determine whether QUE or RES affects acid and bile salt tolerance, the viable counts of tested *Lactobacillus* strains at a specific pH or bile salt concentration in media with or without QUE or RES were compared.

#### Effects on Cell Surface Hydrophobicity

*Lactobacillus* cells grown anaerobically in MRS broth (20–24 h, 37°C, using the AnaeroGen Anaerobic System, Oxoid, Hampshire, United Kingdom) were centrifuged (4500 *g* × 15 min, and 4°C), washed twice and resuspended in PBS with different concentrations (MIC, 1/2 MIC or 1/4 MIC) of QUE or RES to achieve an OD at 560 nm of 1.0, named the A_560_ value (A0).

*n*-Hexadecane (Sigma-Aldrich, St. Louis, MO, United States) was mixed (1:5) with the respective bacterial cell suspension and vortexed for 2 min. After 1 h of incubation at 37°C, the A_560_ value (A) of the formed aqueous layer was measured again. The cell surface hydrophobicity was calculated using the equation:

%H=[(A0-A)/A0]×100

where A0 and A refers to the absorbance values determined before and after the extraction with *n*-hexadecane, respectively (dos Santos et al., 2015). To determine whether QUE or RES affects cell surface hydrophobicity, the cell surface hydrophobicity of *Lactobacillus* strains treated, and not treated with the exposure to different concentrations of QUE or RES was compared.

#### Effects on Autoaggregation and Coaggregation Capacity

For the evaluation of autoaggregation capacity, *Lactobacillus* strains grown anaerobically in MRS broth (20–24 h, 37°C, using the AnaeroGen Anaerobic System, Oxoid, Hampshire, United Kingdom) were harvested by centrifugation (4,500 *g* × 10 min, 20°C), washed, resuspended, and diluted in sterile saline solution (NaCl 0.85 g/100 mL) with different concentrations (i.e., MIC, 1/2 MIC or 1/4 MIC) of QUE or RES to achieve an OD at 660 nm (OD_660_) of 0.3. After 1 h of incubation at 37°C, the OD_660_ value was measured again. The autoaggregation was determined using the equation:

%autoaggregation=[(OD0-OD60)/OD0]×100

where OD0 refers to the initial OD value of the bacterial suspensions, and OD60 refers to the OD value of the bacterial suspension determined after 60 min of incubation (Todorov et al., 2008).

For the evaluation of coaggregation capacity, the *Lactobacillus* strains were similarly grown in MRS broth, harvested by centrifugation (4,500 *g* × 10 min, 20°C), washed, resuspended and diluted in sterile saline solution (NaCl 0.85 g/100 mL) with different concentrations of QUE or RES to achieve an OD_660_ value of 0.3. Next, a 750 μL-aliquot of a suspension of the tested *Lactobacillus* strain was mixed with the same volume of a suspension of the coaggregation bacterial species partner (*L. monocytogenes* INCQS 00266 or *E. coli* INCQS 00219), and vortexed for 30 s. The OD_660_ value of the suspension containing the test *Lactobacillus* strain and the respective bacterial species partner was determined at time zero (baseline–just after mixing the suspensions) and after 60 min of aerobic incubation at 37°C. Coaggregation was measured using the equation:

%coaggregation=[(OD0-OD60)/OD0]×100

where OD0 refers to the initial OD value of the suspension determined at time zero, and OD60 refers to the OD value of the suspension determined after 60 min of incubation (Todorov et al., 2008). To determine whether QUE or RES affects the autoaggregation and coaggregation capacity, the autoaggregation and coaggregation capacity of the examined *Lactobacillus* strains treated and not treated with the exposure to different concentrations of QUE or RES were compared.

#### Effects on Antagonistic Activity Against Pathogens

The antagonistic activity of each *Lactobacillus* strain against the indicator bacterial strains (*L. monocytogenes* INCQS 00266 and *E. coli* INCQS 00219) was evaluated using an agar spot test. The *Lactobacillus* strains were cultivated anaerobically in MRS broth (20–24°C, 37°C, using the AnaeroGen Anaerobic System, Oxoid, Hampshire, United Kingdom), followed by supplementation of the growth medium (MRS both) with different concentrations (MIC, 1/2 MIC, or 1/4 MIC) of QUE or RES. Subsequently, a 10-μL aliquot of the growth medium supplemented with QUE or RES (with viable cell counts in the range of 7–8 log CFU/mL) was spotted on the surface of MRS agar containing 0.2% (w/v) glucose and 1.2% (w/v) bacteriological agar (HiMedia, Mumbai, India) and incubated anaerobically (using the AnaeroGen Anaerobic System, Oxoid, Hampshire, United Kingdom) for 24 h at 37°C. At the end of the incubation period, a 1-mL aliquot of each indicator bacterium suspension was mixed with 18-mL soft BHI agar (with 0.7% agar, w/v) and poured over the spot-inoculated MRS agar. The plates were incubated aerobically at 37°C for 48 h. The antagonistic activity was recorded as the diameters (mm) of growth inhibition zones around each spot. A free growth inhibition zone with a diameter >1 mm (around the spot) was considered as positive inhibitory activity (Jacobsen et al., 1999). MRS agar not inoculated with the test *Lactobacillus* strain and MRS agar without QUE or RES were used as negative controls. To determine whether QUE or RES affects the antagonistic activity, the diameters of the growth inhibition zones of indicator bacterial strains caused by the *Lactobacillus* strains treated and not treated with the exposure to different concentrations of QUE or RES were compared.

#### Effects on Survival Under Simulated Gastrointestinal Conditions

*Lactobacillus* strains grown anaerobically in MRS broth (20–24 h, 37°C, using the AnaeroGen Anaerobic System, Oxoid, Hampshire, United Kingdom) were exposed to simulated gastrointestinal conditions in MRS broth with and without QUE (1/2 MIC) or RES (1/2 MIC) (de Albuquerque et al., 2017; Garcia et al., 2018). Initially, 10 mL-aliquots of MRS broth were placed in glass flasks (50 mL) and inoculated with the tested *Lactobacillus* strain (final viable count in the range of 6–7 log CFU/mL). The simulation of the gastrointestinal conditions was performed continuously in the same flask in phases mimicking mastication, conditions in the esophagus-stomach, duodenum, and ileum. Mechanical agitation was used to simulate the peristaltic movements and the test was performed in an incubator at 37°C with rotation adjustment in each phase. Mastication was simulated using 100 U of α-amylase diluted in 1 mL of 1 mM CaCl_2_, pH adjusted to 6.9 with 1 M NaHCO_3_ and exposure time of 2 min at 200 rpm; esophagus-stomach conditions were simulated with 25 mg of pepsin diluted in 1 mL of 0.1 M HCl, added at a rate of 0.05 mL/mL, with gradually decreasing pH achieved using 1 M HCl (pH 5.5/10 min; pH 4.6/10 min; pH 3.8/10 min; pH 2.8/20 min; pH 2.3/20 min; and pH 2/20 min) under stirring (130 rpm). Duodenal conditions were simulated with 2 g pancreatin/L of 0.1 M NaHCO_3_ and 12 g bovine bile salts/L of 0.1 M NaHCO_3_, pH adjusted to 5 with 0.1 M NaHCO_3_ and an exposure time of 30 min under stirring (45 rpm); the ileal conditions were simulated with pH adjusted to 6.5 using 0.1 M NaHCO_3_ and an exposure time of 60 min under stirring (45 rpm). All the enzymes and bovine bile salts were purchased from Sigma-Aldrich (St. Louis, MO, United States). After each step of the simulated gastrointestinal digestion, a 100 μL-aliquot of inoculated MRS broth with, or without QUE or RES was serially diluted in sterile saline solution (NaCl 0.85 g/100 mL), and plated on MRS agar.

After an incubation period of 24 h at 37°C under anaerobiosis (using the AnaeroGen Anaerobic System, Oxoid, Hampshire, United Kingdom), the viable cells were enumerated and the results were expressed as log CFU/mL. Inoculated MRS broth maintained at 37°C without QUE and RES was used as a control. A detection limit of 1 log CFU/mL was used in these assays. To determine whether QUE or RES affects the survival of the tested *Lactobacillus* strains when exposed to the simulated gastrointestinal digestion, the viable counts of these strains when exposed to each step of the simulated digestion in media with or without QUE or RES were compared.

### Statistical Analysis

All assays were performed in triplicate in three independent experiments, and the results are expressed as the average of the obtained data. Statistical analyses were performed to determine significant differences (*p* ≤ 0.05) among the obtained results using ANOVA, followed by Tukey’s test. The analyses were performed using the software GraphPad Prism 5.0 (San Diego, CA, United States).

## Results

### MIC Determination

Quercetin showed MIC of 1024 μg/mL against *L. plantarum* 49, *L. plantarum* 53, *L. paracasei* 106 and *L. fermentum* 263; and of 512 μg/mL against *L. paracasei* 108 and *L. fermentum* 296. RES showed MIC of 1024 μg/mL against all the examined *Lactobacillus* strains, with the exception of *L. paracasei* 106, for which the MIC was >1024 μg/mL (Table 1).

**TABLE 1:** Minimum inhibitory concentration (MIC) of quercetin (QUE) and resveratrol (RES) on potentially probiotic *Lactobacillus* strains.

	***Lactobacillus* strains**					
	
**Compounds**	***L. plantarum* 49**	***L. plantarum* 53**	***L. paracasei* 106**	***L. paracasei* 108**	***L. fermentum* 263**	***L. fermentum* 296**
QUE	1024 μg/mL	1024 μg/mL	1024 μg/mL	512 μg/mL	1024 μg/mL	512 μg/mL
RES	1024 μg/mL	1024 μg/mL	>1024 μg/mL	1024 μg/mL	1024 μg/mL	1024 μg/mL

### Effects of QUE and RES on Acid and Bile Salt Tolerance

In most cases, the presence of QUE or RES at either of the tested concentrations did not affect (*p* > 0.05) the survival of the examined *Lactobacillus* strains when exposed to pH 7.2 and 5. *L. paracasei* 108 presented lower viable counts (*p* ≤ 0.05) at pH 5 after 1 h in the presence of MIC of QUE when compared to control, as well as after 2 h in the presence of MIC or 1/2 MIC of QUE; the same behavior was observed for *L. paracasei* 108 at pH 5 after 2 h in the presence of 1/2 MIC or 1/4 MIC of RES. *L. paracasei* 106 presented lower (*p* ≤ 0.05) viable counts at pH 5 after 3 h in the presence of either of the tested RES concentrations when compared to the control; similar results were observed for *L. fermentum* 263 after 2 and 3 h in the presence of the MIC of RES or QUE (Supplementary Table S1).

*Lactobacillus plantarum* 53 presented higher viable counts (*p* ≤ 0.05) at pH 2 after 2 h in the presence of the MIC or 1/4 MIC of QUE or RES when compared to the control (Table 2). Similarly, *L. paracasei* 106 and *L. fermentum* 263 presented higher viable counts at pH 2 after 3 h in the presence of QUE or RES at either of the tested concentrations. *L. fermentum* 296 also presented higher viable counts at pH 3 after 2 h in the presence of 1/2 MIC or 1/4 MIC of QUE, as well as at pH 2 after 1 h in the presence of the MIC or 1/2 MIC of QUE when compared to the control. Overall, these data indicated that the presence of QUE or RES was more effective to protect some of the tested *Lactobacillus* strains when exposed to pH 2 or 3.

**TABLE 2:** Viable counts (*n* = 9; average ± standard deviation; log CFU/mL) of potentially probiotic *Lactobacillus* strains when exposed to pH 3 and 2 in media with and without different concentrations of quercetin (QUE) or resveratrol (RES) for different time intervals.

**Strains/treatments**	**pH values/exposure times**					
	
	**pH 3**			**pH 2**		
		
	**1h**	**2 h**	**3 h**	**1 h**	**2 h**	**3 h**
	***L. plantarum* 49**					

MIC QUE	5.6 ± 0.4^*AB*^	6.4 ± 0.2^*A*^	2.5 ± 0.6^*A*^	2.9 ± 0.3^*B*^	4.0 ± 0.5^*A*^	3.3 ± 0.7^*A*^
1/2 MIC QUE	5.4 ± 0.2^*B*^	4.4 ± 0.4^*B*^	2.6 ± 0.5^*A*^	3.5 ± 0.3^*B*^	2.4 ± 0.2^*B*^	2.4 ± 0.4^*A*^
1/4 MIC QUE	6.2 ± 0.2^*A*^	4.5 ± 0.5^*B*^	3.2 ± 0.2^*A*^	3.0 ± 0.3^*B*^	3.6 ± 0.2^*A*^	1.4 ± 0.3^*B*^
MIC RES	5.2 ± 0.2^*B*^	4.4 ± 0.4^*B*^	3.4 ± 0.3^*A*^	4.8 ± 0.4^*A*^	3.8 ± 0.2^*A*^	1.5 ± 0.4^*B*^
1/2 MIC RES	3.3 ± 0.2^*C*^	3.0 ± 0.4^*C*^	3.4 ± 0.3^*A*^	3.6 ± 0.3^*B*^	2.5 ± 0.7^*AB*^	< 1 ± 0.0^*B*^
1/4 MIC RES	5.2 ± 0.2^*B*^	3.2 ± 0.2^*C*^	3.3 ± 0.4^*A*^	4.1 ± 0.4^*AB*^	3.4 ± 0.2^*A*^	2.2 ± 0.4^*A*^
Control	4.5 ± 0.8^*B*^	3.4 ± 0.6^*BC*^	3.0 ± 0.3^*A*^	3.4 ± 0.6^*B*^	3.2 ± 0.3^*A*^	2.4 ± 0.2^*A*^

	***L. plantarum* 53**					

MIC QUE	5.5 ± 0.7^*A*^	3.2 ± 0.2^*B*^	4.1 ± 0.5^*A*^	3.2 ± 0.3^*A*^	4.4 ± 0.5^*A*^	2.5 ± 0.7^*AB*^
1/2 MIC QUE	4.4 ± 0.5^*A*^	3.4 ± 0.6^*AB*^	3.0 ± 0.4^*B*^	2.0 ± 0.7^*B*^	3.3 ± 0.5^*BC*^	1.8 ± 1.1^*B*^
1/4 MIC QUE	3.3 ± 0.4^*B*^	3.6 ± 0.5^*AB*^	3.3 ± 0.4^*AB*^	3.1 ± 0.2^*A*^	3.6 ± 0.3^*AB*^	3.8 ± 0.6^*A*^
MIC RES	4.4 ± 0.3^*A*^	3.4 ± 0.6^*AB*^	2.0 ± 0.5^*C*^	2.0 ± 0.6^*B*^	3.4 ± 0.6^*AB*^	2.2 ± 0.3^*B*^
1/2 MIC RES	3.3 ± 0.5^*B*^	3.2 ± 0.3^*B*^	3.2 ± 0.3^*B*^	2.3 ± 0.4^*B*^	2.0 ± 0.6^*C*^	< 1 ± 0.0^*C*^
1/4 MIC RES	3.3 ± 0.5^*B*^	3.6 ± 0.6^*A*^	< 1 ± 0.0^*D*^	3.4 ± 0.5^*A*^	3.6 ± 0.3^*AB*^	2.6 ± 0.4^*B*^
Control	4.7 ± 0.9^*A*^	4.5 ± 0.4^*A*^	2.5 ± 0.7^*BC*^	2.2 ± 0.2^*B*^	2.0 ± 0.8^*C*^	2.5 ± 0.6^*B*^

	***L. paracasei* 106**					

MIC QUE	3.4 ± 0.9^*A*^	5.0 ± 0.4^*A*^	3.0 ± 0.5^*B*^	3.2 ± 0.6^*BC*^	3.1 ± 0.5^*AB*^	2.4 ± 0.5^*A*^
1/2 MIC QUE	4.1 ± 1.1^*A*^	4.2 ± 0.5^*A*^	4.2 ± 0.2^*A*^	3.4 ± 0.3^*B*^	3.1 ± 0.3^*A*^	2.3 ± 0.5^*A*^
1/4 MIC QUE	3.3 ± 0.4^*A*^	4.1 ± 0.4^*B*^	3.4 ± 0.5^*AB*^	2.2 ± 0.5^*C*^	2.2 ± 0.2^*C*^	1.2 ± 0.3^*B*^
MIC RES	3.2 ± 0.2^*A*^	3.5 ± 0.4^*BC*^	2.9 ± 0.5^*B*^	3.1 ± 0.4^*BC*^	3.4 ± 0.3^*A*^	2.2 ± 0.3^*A*^
1/2 MIC RES	3.2 ± 0.4^*A*^	4.5 ± 0.3^*A*^	4.1 ± 0.2^*A*^	5.5 ± 1.3^*A*^	2.1 ± 1.1^*ABC*^	2.3 ± 0.5^*A*^
1/4 MIC RES	3.3 ± 0.5^*A*^	3.3 ± 0.5^*BC*^	3.2 ± 0.3^*B*^	5.2 ± 1.0^*A*^	2.3 ± 0.4^*BC*^	2.2 ± 0.3^*A*^
Control	2.6 ± 0.4^*A*^	3.1 ± 0.2^*C*^	3.3 ± 0.3^*B*^	3.1 ± 0.4^*BC*^	2.3 ± 0.4^*BC*^	< 1 ± 0.0^*C*^

	***L. paracasei* 108**					

MIC QUE	< 1 ± 0.0	< 1 ± 0.0	< 1 ± 0.0	< 1 ± 0.0	< 1 ± 0.0	< 1 ± 0.0
MIC QUE	< 1 ± 0.0	< 1 ± 0.0	< 1 ± 0.0	< 1 ± 0.0	< 1 ± 0.0	< 1 ± 0.0
1/2 MIC QUE	< 1 ± 0.0	< 1 ± 0.0	< 1 ± 0.0	< 1 ± 0.0	< 1 ± 0.0	< 1 ± 0.0
1/4 MIC QUE	< 1 ± 0.0	< 1 ± 0.0	< 1 ± 0.0	< 1 ± 0.0	< 1 ± 0.0	< 1 ± 0.0
MIC RES	< 1 ± 0.0	< 1 ± 0.0	< 1 ± 0.0	< 1 ± 0.0	< 1 ± 0.0	< 1 ± 0.0
1/2 MIC RES	< 1 ± 0.0	< 1 ± 0.0	< 1 ± 0.0	< 1 ± 0.0	< 1 ± 0.0	< 1 ± 0.0
1/4 MIC RES	< 1 ± 0.0	< 1 ± 0.0	< 1 ± 0.0	< 1 ± 0.0	< 1 ± 0.0	< 1 ± 0.0

	***L. fermentum* 263**					

MIC QUE	6.1 ± 0.4^*A*^	5.4 ± 0.2^*B*^	5.4 ± 0.2^*A*^	5.4 ± 0.3^*A*^	3.5 ± 0.6^*AB*^	2.2 ± 0.4^*A*^
1/2 MIC QUE	6.3 ± 0.6^*A*^	5.6 ± 0.4^*B*^	5.3 ± 0.3^*A*^	3.7 ± 0.4^*B*^	2.7 ± 0.5^*B*^	2.2 ± 0.2^*A*^
1/4 MIC QUE	6.5 ± 0.2^*A*^	5.4 ± 0.3^*B*^	3.2 ± 0.8^*B*^	4.8 ± 0.3^*A*^	1.2 ± 0.3^*C*^	1.2 ± 0.3^*B*^
MIC RES	5.2 ± 0.2^*B*^	6.5 ± 0.3^*A*^	< 1 ± 0.0^*C*^	3.1 ± 0.2^*B*^	3.7 ± 0.4^*A*^	2.3 ± 0.4^*A*^
1/2 MIC RES	6.9 ± 0.5^*A*^	5.3 ± 0.5^*BC*^	4.3 ± 1.1^*AB*^	5.2 ± 0.2^*A*^	4.5 ± 0.5^*A*^	2.9 ± 0.9^*A*^
1/4 MIC RES	4.5 ± 0.2^*C*^	5.2 ± 0.3^*B*^	5.5 ± 0.2^*A*^	3.6 ± 0.5^*B*^	4.2 ± 0.3^*A*^	2.5 ± 0.3^*A*^
Control	6.6 ± 0.2^*A*^	4.4 ± 0.4^*C*^	4.2 ± 0.2^*B*^	5.3 ± 0.4^*A*^	3.7 ± 0.3^*A*^	< 1 ± 0.0^*B*^

	***L. fermentum* 296**					

MIC QUE	6.6 ± 0.4^*A*^	5.6 ± 0.8^*AB*^	2.3 ± 0.5^*B*^	3.7 ± 0.3^*B*^	3.2 ± 0.3^*A*^	1.3 ± 0.4^*Bb*^
1/2 MIC QUE	3.8 ± 1.2^*B*^	6.2 ± 0.2^*A*^	3.9 ± 0.3^*A*^	5.0 ± 0.7^*A*^	4.1 ± 1.1^*A*^	2.1 ± 0.5^*AB*^
1/4 MIC QUE	7.0 ± 0.7^*A*^	6.7 ± 0.3^*A*^	3.0 ± 0.5^*AB*^	3.0 ± 0.5^*BC*^	3.5 ± 0.7^*A*^	1.2 ± 0.3^*B*^
MIC RES	3.7 ± 1.0^*B*^	4.2 ± 0.6^*B*^	3.1 ± 0.5^*AB*^	4.6 ± 0.3^*A*^	3.2 ± 0.2^*A*^	2.4 ± 0.6^*A*^
1/2 MIC RES	6.1 ± 0.2^*A*^	3.9 ± 0.5^*B*^	2.4 ± 0.6^*B*^	2.3 ± 0.5^*C*^	2.0 ± 0.7^*B*^	1.4 ± 0.6^*AB*^
1/4 MIC RES	7.0 ± 0.8^*A*^	4.3 ± 0.3^*B*^	3.1 ± 0.6^*AB*^	2.7 ± 0.7^*BC*^	2.4 ± 0.6^*AB*^	1.1 ± 0.4^*B*^
Control	6.0 ± 1.4^*AB*^	5.0 ± 0.6^*B*^	3.5 ± 0.7^*AB*^	2.4 ± 0.6^*C*^	2.1 ± 0.9^*AB*^	2.5 ± 0.5^*A*^

*Control, 0 μg/mL of QUE or RES. For *L. plantarum* 49, *L. plantarum* 53, and *L. fermentum* 263–MIC of QUE: 1024 μg/mL, 1/2 MIC of QUE: 512 μg/mL, 1/4 MIC of QUE: 256 μg/mL, MIC of RES: 1024 μg/mL, 1/2 MIC of RES: 512 μg/mL, 1/4 MIC of RES: 256 μg/mL; For *L. paracasei* 108 and *L. fermentum* 296–MIC of QUE: 512 μg/mL, 1/2 MIC of QUE: 256 μg/mL, 1/4 MIC of QUE: 128 μg/mL, MIC of RES: 1024 μg/mL, 1/2 MIC of RES: 512 μg/mL, 1/4 MIC of RES: 256 μg/mL; For *L. paracasei* 106–MIC of QUE: 1024 μg/mL, 1/2 MIC of QUE: 512 μg/mL, 1/4 MIC of QUE: 256 μg/mL, MIC of RES: 1024 μg/mL (assumed), 1/2 MIC of RES: 512 μg/mL (assumed), 1/4 MIC of RES: 256 μg/mL (assumed). ^*A,B*^Different superscript capital letters in the same column denote difference (*p* ≤ 0.05) among the counts for the tested *Lactobacillus* strain when exposed to a pH in media with or without QUE or RES, based on Tukey’s test.*

Specifically, *L. plantarum* 49 presented higher viable counts (*p* ≤ 0.05) in the presence of 1% bile salts after 3 h of exposure to 1/2 MIC or 1/4 MIC of QUE, as well as to the MIC or 1/4 MIC of RES when compared to the control. In contrast, *L. paracasei* 108 presented lower viable counts (*p* ≤ 0.05) in the presence of 0.3% bile salts after 1 h of exposure to QUE at either of the tested concentrations when compared to the control (Supplementary Table S2). Overall, QUE and RES at either of the tested concentrations did not affect (*p* > 0.05) the viable counts of the examined *Lactobacillus* strains when exposed to 0.15, 0.3 or 1% bile salts during the 3 h of exposure.

### Effects of QUE and RES on Cell Surface Hydrophobicity

The cultivation of *L. paracasei* 106, *L. paracasei* 108, *L. fermentum* 263 and *L. fermentum* 296 in the presence of either of the tested QUE concentrations induced increased cell surface hydrophobicity (*p* ≤ 0.05) when compared to control. An increase (*p* ≤ 0.05) in cell surface hydrophobicity of *L. plantarum* 49 and *L. plantarum* 53 was induced by the MIC of QUE, while the MIC and 1/2 MIC of QUE increased the cell surface hydrophobicity of *L. plantarum* 53. All tested concentrations of RES increased (*p* ≤ 0.05) the cell surface hydrophobicity of *L. fermentum* 296, and the 1/2 MIC of RES increased the cell surface hydrophobicity of *L. paracasei* 106 and *L. paracasei* 108. However, 1/4 MIC of QUE and all tested concentrations of RES reduced (*p* ≤ 0.05) the cell surface hydrophobicity of *L. plantarum* 49, and 1/2 MIC and/or 1/4 MIC of RES reduced the cell surface hydrophobicity of *L. plantarum* 53 and *L. paracasei* 108. All tested concentrations of RES did not affect (*p* > 0.05) the cell surface hydrophobicity of *L. fermentum* 263 (Table 3).

**TABLE 3:** Effects of different concentrations of quercetin (QUE) and resveratrol (RES) on cell surface hydrophobicity, autoaggreation, and coaggregation properties (*n* = 9; average ± standard deviation) of potentially probiotic *Lactobacillus* strains.

**Treatments**	**Hydrophobi city (%)**	**Auto aggregation (%)**	**Coaggregation (%)**	
			
			***L. monocytogenes***	***E. coli***
	***L. plantarum* 49**			

MIC QUE	52.5 ± 3.5^∗^	51.2 ± 3.1^∗^	70.0 ± 0.7^∗^	74.8 ± 3.9^∗^
1/2 MIC QUE	22.0 ± 2.8	22.3 ± 3.3^∗^	54.7 ± 6.6^∗^	50.0 ± 2.9^∗^
1/4 MIC QUE	14.0 ± 1.4^∗^	13.8 ± 1.1^∗^	55.6 ± 7.9^∗^	50.4 ± 2.0^∗^
MIC RES	8.9 ± 1.6^∗^	10.6 ± 2.0	31.7 ± 2.3^∗^	38.6 ± 1.9^∗^
1/2 MIC RES	7.3 ± 0.1^∗^	17.0 ± 1.4^∗^	31.2 ± 1.1^∗^	32.9 ± 0.5^∗^
1/4 MIC RES	14.1 ± 1.6^∗^	13.7 ± 1.8^∗^	21.4 ± 2.3	29.0 ± 1.4^∗^
Control	19.1 ± 1.3	9.2 ± 1.2	15.0 ± 4.2	19.0 ± 1.4

	***L. plantarum* 53**			

MIC QUE	44.0 ± 5.7^∗^	41.0 ± 2.8^∗^	51.5 ± 2.1^∗^	28.5 ± 2.1^∗^
1/2 MIC QUE	46.1 ± 1.5^∗^	29.5 ± 0.7^∗^	61.1 ± 7.2^∗^	18.5 ± 2.1^∗^
1/4 MIC QUE	29.0 ± 1.4	22.5 ± 3.5	58.5 ± 7.8^∗^	5.4 ± 2.3^∗^
MIC RES	25.8 ± 1.1	22.6 ± 1.9	48.8 ± 1.7^∗^	7.0 ± 2.1^∗^
1/2 MIC RES	17.0 ± 4.2^∗^	35.8 ± 1.1^∗^	28.5 ± 3.4^∗^	20.3 ± 3.2^∗^
1/4 MIC RES	9.8 ± 1.1^∗^	25.8 ± 0.3	19.9 ± 0.2	17.1 ± 3.0^∗^
Control	26.7 ± 2.4	25.0 ± 1.3	17.3 ± 0.9	2.5 ± 0.1

	***L. paracasei* 106**			

MIC QUE	53.6 ± 5.0^∗^	42.5 ± 3.5	71.1 ± 1.6^∗^	68.0 ± 2.8^∗^
1/2 MIC QUE	43.6 ± 5.1^∗^	30.9 ± 1.3	65.0 ± 4.2^∗^	56.6 ± 2.3^∗^
1/4 MIC QUE	39.0 ± 1.4^∗^	33.5 ± 2.1	71.3 ± 4.7^∗^	31.1 ± 5.5
MIC RES	30.7 ± 0.9	36.6 ± 2.3	62.3 ± 6.1^∗^	42.3 ± 3.3
1/2 MIC RES	46.5 ± 3.5^∗^	33.3 ± 1.1	40.7 ± 6.1	37.3 ± 0.9
1/4 MIC RES	28.5 ± 2.1	31.5 ± 4.9	28.4 ± 0.6	39.2 ± 1.8
Control	29.8 ± 2.9	38.5 ± 9.2	26.1 ± 8.4	38.7 ± 3.6

	***L. paracasei* 108**			

MIC QUE	48.5 ± 2.1^∗^	49.8 ± 2.5^∗^	41.5 ± 2.1^∗^	57.0 ± 2.9^∗^
1/2 MIC QUE	46.9 ± 1.3^∗^	31.8 ± 4.5	35.4 ± 0.9^∗^	62.2 ± 3.1^∗^
1/4 MIC QUE	27.1 ± 4.1^∗^	23.0 ± 4.2^∗^	30.6 ± 2.1^∗^	39.3 ± 1.8^∗^
MIC RES	9.9 ± 1.6	47.7 ± 0.5^∗^	38.7 ± 2.4^∗^	33.0 ± 4.2^∗^
1/2 MIC RES	23.2 ± 2.5^∗^	17.2 ± 3.9^∗^	11.6 ± 2.2	33.1 ± 1.3^∗^
1/4 MIC RES	9.2 ± 1.1^∗^	22.0 ± 2.8^∗^	12.3 ± 2.5	37.9 ± 4.1^∗^
Control	13.8 ± 1.7	38.0 ± 2.9	11.6 ± 1.9	22.7 ± 1.9

	***L. fermentum* 263**			

MIC QUE	37.5 ± 3.5^∗^	39.0 ± 1.4^∗^	40.0 ± 2.8^∗^	43.0 ± 1.4^∗^
1/2 MIC QUE	26.8 ± 1.7^∗^	36.5 ± 2.2^∗^	47.0 ± 4.2^∗^	39.3 ± 1,0^∗^
1/4 MIC QUE	18.6 ± 1.9^∗^	21.1 ± 4.1	52.8 ± 3.9^∗^	39.7 ± 2.5^∗^
MIC RES	10.9 ± 1.6	21.1 ± 1.6	45.2 ± 3.1^∗^	46.2 ± 3.1^∗^
1/2 MIC RES	8.2 ± 1.2	20.0 ± 2.8	37.2 ± 3.1^∗^	36.9 ± 2.7^∗^
1/4 MIC RES	8.2 ± 1.1	17.0 ± 2.8	33.4 ± 3.5	19.9 ± 1.3
Control	8.9 ± 2.9	17.5 ± 6.4	23.5 ± 2.1	21.0 ± 2.9
MIC QUE	32.1 ± 1.2^∗^	31.7 ± 2.5^∗^	79.9 ± 2.7^∗^	68.5 ± 2.2^∗^
1/2 MIC QUE	26.1 ± 1.2^∗^	32.1 ± 3.0^∗^	33.0 ± 4.3^∗^	34.1 ± 1.3
1/4 MIC QUE	29.4 ± 2.0^∗^	26.2 ± 1.1^∗^	22.2 ± 9.7	30.6 ± 0.8
MIC RES	11.1 ± 1.3^∗^	12.6 ± 3.4	29.1 ± 1.3	40.7 ± 3.8^∗^

	***L. fermentum* 296**			

1/2 MIC RES	9.9 ± 1.1^∗^	15.9 ± 0.6	25.2 ± 1.1	32.1 ± 2.9
1/4 MIC RES	13.8 ± 2.5^∗^	15.0 ± 1.4	23.5 ± 2.1	18.9 ± 5.8
Control	5.6 ± 1.5	17.8 ± 3.1	18.9 ± 4.8	29.8 ± 5.2

*Control, 0 μg/mL of QUE or RES. For *L. plantarum* 49, *L. plantarum* 53, and *L. fermentum* 263–MIC of QUE: 1024 μg/mL, 1/2 MIC of QUE: 512 μg/mL, 1/4 MIC of QUE: 256 μg/mL, MIC of RES: 1024 μg/mL, 1/2 MIC of RES: 512 μg/mL, 1/4 MIC of RES: 256 μg/mL; For *L. paracasei* 108 and *L. fermentum* 296–MIC of QUE: 512 μg/mL, 1/2 MIC of QUE: 256 μg/mL, 1/4 MIC of QUE: 128 μg/mL, MIC of RES: 1024 μg/mL, 1/2 MIC of RES: 512 μg/mL, 1/4 MIC of RES: 256 μg/mL; For *L. paracasei* 106–MIC of QUE: 1024 μg/mL, 1/2 MIC of QUE: 512 μg/mL, 1/4 MIC of QUE: 256 μg/mL, MIC of RES: 1024 μg/mL (assumed), 1/2 MIC of RES: 512 μg/mL (assumed), 1/4 MIC of RES: 256 μg/mL (assumed).^∗^, means difference (*p* ≤ 0.05) of the measured property in the tested strain in comparison to the control, based on Tukey’s test.*

### Effects of QUE and RES on Autoaggregation and Coaggregation

No decrease (*p* > 0.05) in autoaggregation capacity of tested *Lactobacillus* strains was caused by either of the tested concentrations of QUE and RES, with the exception of *L. paracasei* 108 that presented decreased (*p* ≤ 0.05) autoaggregation capacity when exposed to 1/4 MIC of QUE and 1/2 MIC or 1/4 MIC of RES when compared to control. All tested concentrations of QUE increased (*p* ≤ 0.05) the autoaggregation capacity of *L. plantarum* 49 and *L. fermentum* 296, and the MIC and 1/2 MIC of QUE increased the autoaggregation capacity of *L. plantarum* 53 and *L. fermentum* 263. 1/2 MIC of RES increased the autoaggregation capacity of *L. plantarum* 49 and *L. plantarum* 53. All tested concentrations of QUE and RES did not affect (*p* > 0.05) the autoaggregation capacity of *L. paracasei* 106. All tested concentrations of RES did not affect the autoaggregation capacity of *L. fermentum* 263 and *L. fermentum* 296, as well as the MIC and/or 1/4 MIC of RES did not affect this property in *L. plantarum* 49 and *L. plantarum* 53 (Table 3).

No decrease in coaggregation capacity was caused by either of the tested concentrations of QUE and RES. All tested concentrations of QUE increased (*p* ≤ 0.05) the capacity of *L. plantarum* 49, *L. plantarum* 53 and *L. paracasei* 108 to coaggregate with *L. monocytogenes* and/or *E. coli*. All tested concentrations of RES increased the capacity of *L. plantarum* 49, *L. plantarum* 53 and *L. paracasei* 108 to coaggregate with *E. coli*, as well as the MIC and 1/2 MIC of RES increased the capacity of *L. plantarum* 49, *L. plantarum* 53 and *L. fermentum* 263 to coaggregate with *L. monocytogenes* (Table 3).

### Effects of QUE and RES on Antagonistic Activity Against Pathogens

In most cases, the presence of QUE or RES did not affect (*p* ≤ 0.05) the antagonistic activity of the *Lactobacillus* strains against *L. monocytogenes* and *E. coli*. In particular, the presence of the MIC and/or 1/2 MIC of QUE increased (*p* ≤ 0.05) the antagonistic activity of *L. plantarum* 53 against *L. monocytogenes* and *E. coli*; of *L. paracasei* 106 against *L. monocytogenes*; and of *L. paracasei* 108 against *E. coli*. All tested concentrations of QUE and 1/2 MIC and 1/4 MIC of RES increased (*p* ≤ 0.05) the antagonistic activity of *L. fermentum* 296 against *L. monocytogenes* (Supplementary Table S3).

### Effects of QUE and RES on Survival Under Simulated Gastrointestinal Conditions

**FIGURE 1:**
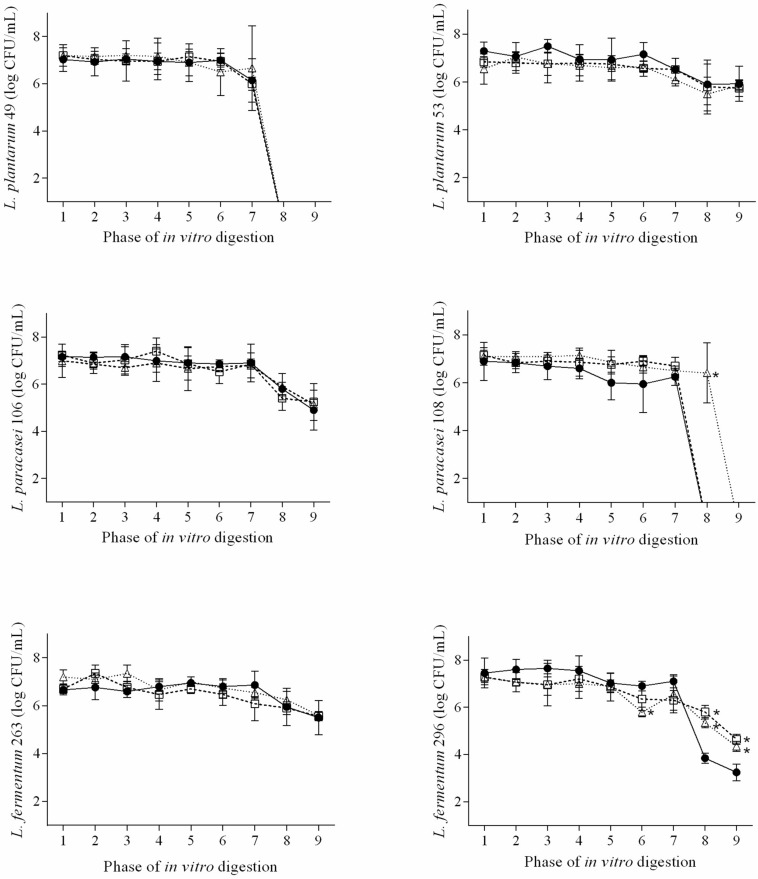
Viable cell counts (*n* = 9; average ± standard deviation; log CFU/mL) of *L. plantarum* 49, *L. plantarum* 53, *L. paracasei* 106, *L. paracasei* 108, *L. fermentum* 263, and *L. fermentum* when exposed to the different phases of a simulated gastrointestinal digestion in de Mann, Rogosa and Sharpe (MRS), MRS with 1/2 MIC of QUE (MRS + QUE), and MRS with 1/2 MIC of RES (MRS + RES). The error bars represent the standard deviations. Phase 1, mouth conditions, pH 6.9, and exposure time 2 min; phase 2, esophagus-stomach conditions, pH 5.5, and exposure time 12 min; phase 3, esophagus-stomach conditions, pH 4.6, and exposure time 22 min; phase 4, stomach conditions, pepsin, pH 3.8, and exposure time 32 min; phase 5, stomach conditions, pepsin, pH 2.8, and exposure time 52 min; phase 6, stomach conditions, pepsin, pH 2.3, and exposure time 72 min; phase 7, stomach conditions, pepsin, pH 2, and exposure time 92 min; phase 8, duodenum conditions, pancreatin + bile salts, pH 5, and exposure time 122 min; and phase 9, ileum conditions, pH 6.5, and exposure time 182 min. ^∗^, means difference (*p* ≤ 0.05) of the viable cell count of the tested strain in the respective phase of *in vitro* digestion when compared to the control, based on Tukey’s test.

The decreases in viable counts of the examined *Lactobacillus* strains were always of <2 log CFU/mL up to the 7th phase of the *in vitro* digestion regardless the presence of QUE or RES (Figure 1). *L. plantarum* 49 presented viable counts of <1 log CFU/mL when exposed to the 8th (duodenal conditions) and 9th phase (ileal conditions) of the *in vitro* digestion in media with and without QUE or RES. Counts of <1 log CFU/mL were also observed for *L. paracasei* 108 when exposed to the 8th and 9th phases of the *in vitro* digestion in medium with QUE, as well as when exposed to the 9th phase in medium with RES. The counts of *L. plantarum* 53, *L. paracasei* 106, *L. plantarum* 263, and *L. plantarum* 296 when exposed to the 8th and 9th phases of the *in vitro* digestion in media with QUE or RES were in the range of 4.4–6.4 log CFU/mL.

The presence of QUE or RES overall did not affect (*p* > 0.05) the ability of the examined *Lactobacillus* strains to survive the exposure to the different phases of the *in vitro* digestion. However, the counts of *L. paracasei* 108 when exposed to the 8th phase of the *in vitro* digestion were higher (*p* ≤ 0.05) in medium with RES than in medium without RES. Higher counts (*p* ≤ 0.05) were also observed for *L. plantarum* 296 when exposed to the 8th and 9th phases of the *in vitro* digestion in media with QUE or RES.

## Discussion

Quercetin and resveratrol presented high MIC values (≥512 μg/mL) against all the six examined *Lactobacillus* strains, which are indicative of low inhibitory effects on target strains (van Vuuren, 2008; Diniz-Silva et al., 2017). The available literature has shown variable results considering the effects of polyphenols and phenolic-rich extracts on the growth of lactic acid bacteria. An early study reported no inhibitory effects of phenolic-rich extracts of spices and medicinal plants (313–2500 μg/mL) against *Lactobacillus* species (Chan et al., 2018), as well as of caffeic acid, gallic acid, tannic acid, catechin, epicatechin and QUE (5000 μg/disk) against *Lactobacillus acidophilus* CECT 903 (Hervert-Hernández et al., 2009). Furthermore, polyphenol/anthocyanin-rich ethanol red fruit extract (0.24–250 mg/mL) has shown stimulatory effects on the growth of *Lactobacillus rhamnosus* IMC 501 and *L. paracasei* IMC 502 (Coman et al., 2018), while wine polyphenols have shown to protect the viability of different lactic acid bacteria in a probiotic formulation (Llano et al., 2017).

However, an early study reported that flavan-3-ol-enriched grape seed extract (1 mg/mL) inhibited the growth of *L. plantarum*, *L. casei* and *L. bulgaricus*. These inhibitory effects were associated with the high amounts of gallate-derived compounds [e.g., (−)-epicatechin-3-O-gallate] in the grape seed extract (Tabasco et al., 2011). The inhibitory effects exerted by some phenolic compounds on specific bacterial species have been related to their ability to cause alterations in structure of cytoplasmatic membrane with changes in polarization and permeability (Mora-Pale et al., 2015; Wang et al., 2017).

Concentrations referring to MIC, 1/2 MIC and 1/4 MIC of QUE and RES were investigated to their effects on probiotic-related *in vitro* properties of the examined *Lactobacillus* strains. The doses of QUE and RES used in this study (87.5–1024 mg/mL) are among reported mean values for estimated daily polyphenols intake (Taguchi et al., 2015; Karam et al., 2018), as well as for use of QUE and RES as daily dietary supplements (Egert et al., 2008; Tome-Carneiro et al., 2013).

The tolerance to acidic pH and bile salts are important characteristics of probiotics (Monteagudo-Mera et al., 2012; de Albuquerque et al., 2017). The results of this study showed that QUE and RES exerted no influence on the survival of the tested *Lactobacillus* strains at pH 7.2, but at some tested concentrations these compounds caused decreases in the survival of three (*L. paracasei* 106, *L. paracasei* 108, and *L. fermentum* 263) of the six examined strains when exposed to pH 5. In contrast, QUE and RES, at some tested concentrations, increased the survival of *L. paracasei* 106, *L. fermentum* 263, and/or *L. fermentum* 296 when exposed to pH 2 or 3 after at least one of the measured exposure time intervals (1–3 h), indicating that these polyphenols could exert protective effects on some probiotics in more acidic environments. It has been suggested that food components, including polyphenols, could act as buffers in acidic environments protecting probiotics against pH values as low as 2 and 3 (Monteagudo-Mera et al., 2012). In disagreement with the findings of this study, catechin (0.3%) and gallic acid (0.8%) decreased the survival of the starter lactic acid culture *Streptococcus thermophilus* CHCC 3534 at pH 2 and/or 3. In the same study, catechin and gallic acid increased and decreased the survival of *S. thermophilus* CHCC 3534 in the presence of 0.4% bile salts, respectively (Khalil, 2010).

All tested concentrations of QUE increased the cell surface hydrophobicity of *L. paracasei* 106, *L. paracasei* 108, *L. fermentum* 263 and *L. fermentum* 296 when measured by adhesion to *n*-hexadecane. Evaluation of probiotics adhesion to *n*-hexadecane is considered a valid qualitative phenomenological approach to estimate the ability to adhere to epithelial cells (Kiely and Olson, 2000; dos Santos et al., 2014), even though it is not a prerequisite for strong adherence because the microbial adhesion to host tissue involves several mechanisms (dos Santos et al., 2015). Cell surface hydrophobicity has been also related to the prevention of pathogen adhesion to intestinal cells by probiotics (dos Santos et al., 2015; de Souza E.L. et al., 2019). In most cases, QUE and RES caused improvements in the autoaggregation and coaggregation capacity of the examined *Lactobacillus* strains. Aggregation is an important feature for biofilm formation by probiotic bacteria, assisting them in adhering to intestinal mucosa (de Souza E.L. et al., 2019). The maintenance or improvement of the capacity of probiotics to coaggregate with pathogens should be considered a positive feature of QUE and RES, since it has been cited as one of the initial steps to prevent colonization of the host gastrointestinal tract by pathogens (Todorov et al., 2008). Together aggregation and coaggregation enable probiotics to better compete with pathogens for host binding sites (Ferreira et al., 2011).

Few studies have assessed the effects of polyphenols on the adhesion capacity of probiotics. Apple pulp extract (10 and 20 mg/mL) decreased the adhesion capacity of *Lactobacillus grasseri* R to Caco-2 cells, while apple peel extract (10 and 20 mg/mL) and QUE (20 μg/mL) increased the adhesion capacity of *L. grasseri* R and *L. casei* FMP (Volstatova et al., 2017). In addition, the adaptation of *S. thermophilus* CHCC 3534 in media with catechin (0.3%) resulted in decreased bacterial adherence (Khalil, 2010). Both cell surface hydrophobicity and aggregation are strongly related to the ability of probiotics to adhere to intestinal mucosa (Deepika et al., 2012), which reinforces the importance of the overall positive modulatory effects exerted by QUE and RES on these physiological features in *Lactobacillus* strains used in this study. Although previous studies have shown variable results concerning the effects of polyphenols on adhesion properties of probiotics (Khalil, 2010; Bustos et al., 2012; Volstatova et al., 2017), it has been suggested that polyphenols could increase the adhesion capacity of specific probiotic strains by inducing biosynthesis or secretion of multifunction proteins (named moonlighting proteins; e.g., glycolytic enzyme pyruvate kinase) engaged in adhesion of bacteria to epithelial cells (Celebioglu et al., 2018).

Quercetin and resveratrol presented overall little influence on the antagonistic activity of the examined *Lactobacillus* species against *L. monocytogenes* and *E. coli*. Specifically, the antagonistic activities of *L. plantarum* 53 against *L. monocytogenes* and *E. coli*, as well as of *L. paracasei* 108 against *E. coli* were increased by the MIC and 1/2 MIC of QUE, while the antagonistic activity of *L. fermentum* 296 against *L. monocytogenes* were increased by all tested concentrations of QUE and 1/2 MIC and 1/4 MIC of RES. Only one previous study reported that the combined use of a commercial phenolic-rich green tea extract with probiotics caused increased inhibitory effects on *Staphylococcus aureus* and *Streptococcus pyogenes*. The authors stated that the occurrence of enhanced inhibitory effects on pathogens from the interaction of *Lactobacillus* and phenolic compounds could be due to additional mechanisms offered by the latter (e.g., membrane depolarization and altered membrane permeability) to those exerted by antimicrobial metabolites commonly produced by these bacteria (e.g., bacteriocins, peptides, and organic acids) to act on target cells (Su et al., 2008).

QUE and RES exerted no negative impact on the survival of the examined *Lactobacillus* strains when exposed to simulated *in vitro* digestion. Specifically, QUE and RES increased the survival of *L. paracasei* 108 when exposed to the duodenal conditions. Early studies have reported that phenolic-rich matrices could protect probiotic *Lactobacillus* strains during the passage through the gastrointestinal tract. Grape marc protected *L. plantarum* 12A, *L. plantarum* PU1, *L. paracasei* 14A and *Bifidobacterium breve* 15A when exposed to a stomach-mimicking condition (Campanella et al., 2017) and mashed tomato protected *Lactobacillus reuteri* ATCC 55730 when exposed to simulated gastrointestinal conditions (García-Hernández et al., 2018). These possible protective effects of phenolic compounds have been primarily attributed to their antioxidant properties, which could protect probiotic cells from the damage caused by exposure to the harsh conditions found in the gastrointestinal tract (Maukonen and Saarela, 2015). The fact that QUE and RES did not exert any negative impact on the survival of the potentially probiotic *Lactobacillus* strains is an interesting finding because only the bioactive components that resist the stomach and small intestine conditions can reach the large intestine and exert their beneficial effects on the host (García-Hernández et al., 2018).

The different effects exerted by QUE and RES on the measured *in vitro* properties of the examined *Lactobacillus* strains could be associated with the specific hydroxylation pattern of these polyphenols. The number and position of hydroxyl groups in the phenolic ring seem to be associated with the importance of different biological effects exerted by polyphenols (Farhadi et al., 2016; de Souza E.L. et al., 2019). QUE presents more hydroxyl groups than RES, which could be responsible for the strongest protective effects overall exerted by the former on some of the measured *in vitro* properties in examined *Lactobacillus* strains. Considering the responses of the tested *Lactobacillus* strains, the overall ranking of the strains that presented the better responses to QUE and RES in the measured *in vitro* properties were *L. fermentum* 263/*L. fermentum* 296 > *L. plantarum* 49/*L. plantarum* 53 > *L. paracasei* 106/*L. paracasei* 108. However, the obtained results did not show a clear relation of the tested concentrations of QUE or RES (MIC, 1/2 MIC and 1/4 MIC) to the size of the observed effects on the measured *in vitro* properties of the examined *Lactobacillus* strains.

It is noteworthy to consider that results concerning the effects exerted by QUE and RES on the measured *in vitro* physiological properties of tested potentially probiotic *Lactobacillus* strains present limitations to be extrapolated to *in vivo* conditions, since a variety of factors present in gastrointestinal environment (e.g., dietary factors, number and types of microorganisms, peristatic flow, cell-to-cell communication, and individual host response) could modify the expected bacterial responses to these polyphenols and, consequently, their effects on the host (Lebeer et al., 2008; Bustos et al., 2012). However, results of *in vitro* experiments have been considered important tools to indicate the effects of phenolic-rich foods and beverages, phenolic rich-extracts and individual phenolic compounds on the functionalities of probiotic microorganisms (Khalil, 2010; Bustos et al., 2012; Deepika et al., 2012; Volstatova et al., 2017).

The results of this study showed that QUE and RES presented low inhibitory effects on different potentially probiotic *Lactobacillus* strains, being necessary high concentrations of these polyphenols to cause growth inhibition. The presence of QUE and RES (MIC, 1/2 MIC and 1/4 MIC) mostly exerted no influence or improved the different measured *in vitro* probiotic-related properties of the examined *Lactobacillus* strains, as well as their ability to survive experimental conditions mimicking the gastrointestinal digestion. In some conditions, the presence of QUE or RES decreased the cell surface hydrophobicity and the ability to survive moderate pH in some of the tested *Lactobacillus* strains. When the presence of QUE or RES resulted in increased or decreased performance in the measured functionality-related properties, the effects varied relative to the type of polyphenol, concentration assayed and *Lactobacillus* strain tested. Overall, QUE exerted better protective effects than RES on the measured *in vitro* properties in tested *Lactobacillus* strains, and tested *L. fermentum* and *L. plantarum* strains presented the better responses in the measured *in vitro* properties when treated with QUE or RES. These results indicate that the combined use of QUE or RES with probiotic *Lactobacillus* strains could result in some advantage with respect to the potential beneficial effects exerted on the host. However, the concentration of these compounds should be cautiously considered to achieve these desirable effects and seems to vary according to the probiotic species/strain selected. Further studies to clarify the mechanisms underlying the effects of QUE and RES on probiotic-related properties of *Lactobacillus* species/strains will help to gain a better insight into the interaction of these polyphenols on beneficial microorganisms forming the complex gut microbiota and their potential outcomes on host health.

## Data Availability Statement

All datasets generated for this study are included in the manuscript/Supplementary Files.

## Author Contributions

AS and TA performed the experiments. AS and TA analyzed the data and prepared the first draft of the manuscript. ES and JB conceptualized and coordinated the study and participated in its design, and wrote the final manuscript.

## Conflict of Interest

The authors declare that the research was conducted in the absence of any commercial or financial relationships that could be construed as a potential conflict of interest.

## References

[B1] Abushelaibi A. Al-Mahadin S. El-Tarabily K. Shah N. P. Ayyash M. (2017). Characterization of potential probiotic lactic acid bacteria isolated from camel milk. *LWT Food Sci. Technol.* 79 316–325. 10.1016/j.lwt.2017.01.041 30147735

[B2] Bustos I. Garcia-Cayuela T. Hernaìndez-Ledesma B. Pelaìez C. Requena T. Martínez-Cuesta M. C. (2012). Effect of flavan-3-ols on the adhesion of potential probiotic lactobacilli to intestinal cells. *J. Agric. Food Chem.* 60 9082–9088. 10.1021/jf301133g 22889010

[B3] Campanella D. Rizzello C. G. Fasciano C. Gambacorta G. Pinto D. Marzani B. (2017). Exploitation of grape marc as functional substrate for lactic acid bacteria and bifidobacteria growth and enhanced antioxidant activity. *Food Microbiol.* 65 25–35. 10.1016/j.fm.2017.01.019 28400010

[B4] Celebioglu H. U. Delsoglio M. Brix S. Pessione E. Svensson B. (2018). Plant polyphenols stimulate adhesion to intestinal mucosa and induce proteome changes in the probiotic *Lactobacillus acidophilus* NCFM. *Mol. Nutr. Food Res.* 62:1700638. 10.1002/mnfr.201700638 29205785

[B5] Chan C. L. Gan R. Y. Shah N. P. Corke H. (2018). Polyphenols from selected dietary spices and medicinal herbs differentially affect common food-borne pathogenic bacteria and lactic acid bacteria. *Food Cont.* 92 437–443. 10.1016/j.foodcont.2018.05.032

[B6] CLSI (2012). *Performance Standards for Antimicrobial Susceptibility Testing; Twenty-second Informational Supplement (CLSI M100–S22).* Wayne, PA: Clinical and Laboratory Standards Institute.

[B7] Coman M. M. Oancea A. M. Verdenelli M. C. Cecchini C. Bahrim G. E. Orpianesi C. (2018). Polyphenol content and *in vitro* evaluation of antioxidant, antimicrobial and prebiotic properties of red fruit extracts. *Eur. Food Res. Technol.* 244 735–745. 10.1007/s00217-017-2997-9

[B8] Cueva C. Gil-Sánchez I. Ayuda-Durán B. González-Manzano S. González-Paramás A. M. Santos-Buelga C. (2017). An integrated view of the effects of wine polyphenols and their relevant metabolites on gut and host health. *Molecules* 22:99. 10.3390/molecules22010099 28067835 PMC6155716

[B9] David L. A. Maurice C. F. Button J. E. Turnbaugh P. J. (2014). Diet rapidly and reproducibly alters the human gut microbiome. *Nature* 505 559–563. 10.1038/nature12820 24336217 PMC3957428

[B10] de Albuquerque T. M. R. Garcia E. F. de Oliveira Araújo A. Magnani M. Saarela M. de Souza E. L. (2017). *In vitro* characterization of *Lactobacillus* strains isolated from fruit processing by-products as potential probiotics. *Probiotics Antimicrob. Proteins* 10 704–716. 10.1007/s12602-017-9318-2 28836171

[B11] de Souza B. M. S. Borgonovi T. F. Casarotti S. N. Todorov S. D. Penna A. L. B. (2019). *Lactobacillus case*i and *Lactobacillus fermentum* strains isolated from mozzarella cheese: probiotic potential, safety, acidifying kinetic parameters and viability under gastrointestinal tract conditions. *Probiotics Antimicrob. Proteins* 11 382–396. 10.1007/s12602-018-9406-y 29542032

[B12] de Souza E. L. de Albuquerque T. M. R. dos Santos A. S. Massa N. M. L. de Brito-Alves J. L. (2019). Potential interactions among phenolic compounds and probiotics for mutual boosting of their health-promoting properties and food functionalities - A review. *Crit. Rev. Food. Sci. Nutr.* 59 1645–1659. 10.1080/10408398.2018.1425285 29377718

[B13] Deepika G. Rastall R. A. Charalampopoulos D. (2012). Effect of food models and low-temperature storage on the adhesion of *Lactobacillus rhamnosus* GG to Caco-2 cells. *J. Agric. Food Chem.* 59 8661–8666. 10.1021/jf2018287 21756003

[B14] Diniz-Silva H. T. Magnani M. Siqueira S. de Souza E. L. de Siqueira-Júnior J. P. (2017). Fruit flavonoids as modulators of norfloxacin resistance in *Staphylococcus aureus* that overexpresses norA. *LWT Food Sci. Technol.* 85 324–326. 10.1016/j.lwt.2016.04.003

[B15] Dolinsky V. W. Chakrabarti S. Pereira T. J. Oka T. Zordoky B. N. Morton J. S. (2013). Resveratrol prevents hypertension and cardiac hypertrophy in hypertensive rodents. *Can. J. Diabetes* 37:S23 10.1016/j.jcjd.2013.08.070 23707558

[B16] dos Santos K. M. O. Vieira A. D. S. Buriti F. C. A. do Nascimento J. C. F. de Melo M. E. S. Bruno L. M. (2015). Artisanal Coalho cheeses as source of beneficial *Lactobacillus plantarum* and *Lactobacillus rhamnosus* strains. *Dairy Sci. Technol.* 95 209–230. 10.1007/s13594-014-0201-6

[B17] dos Santos K. M. O. Vieira A. D. S. Salles H. O. Oliveira J. S. Rocha C. R. C. Bruno L. M. (2014). Safety, beneficial and technological properties of *Enterococcus faecium* isolated from Brazilian cheeses. *Braz. J. Microbiol.* 46 237–249. 10.1590/S1517-838246120131245 26221113 PMC4512068

[B18] Egert S. Woffram S. Bosy-Westphal A. Boesch-Saadatmandi C. Wagner A. E. Geral J. F. (2008). Daily quercetin supplementation dose-dependently increases plasma quercetin concentrations in healthy humans. *J. Nutr.* 138 1615–1621. 10.1093/jn/138.9.1615 18716159

[B19] Farhadi K. Esmaeilzadeh F. Hatami M. Forough M. Molaie R. (2016). Determination of phenolic compounds content and antioxidant activity in skin, pulp, seed, cane and leaf of five native grape cultivars in West Azerbaijan province, Iran. *Food Chem.* 199 847–855. 10.1016/j.foodchem.2015.12.083 26776043

[B20] Ferreira C. L. Grześkowiak L. Collado M. C. Salminen S. (2011). *In vitro* evaluation of *Lactobacillus gasseri* strains of infant origin on adhesion and aggregation of specific pathogens. *J. Food Prot.* 74 1482–1487. 10.4315/0362-028X 21902917

[B21] Garcia E. F. Luciano W. A. de Albuquerque T. M. R. Arcanjo N. M. O. Madruga M. S. Lima M. S. (2018). The performance of five fruit-derived and freeze-dried potentially probiotic *Lactobacillus* strains in apple, orange and grape juices. *J. Sci. Food Agric.* 98 5000–5010. 10.1002/jsfa.9034 29602227

[B22] Garcia E. F. Luciano W. A. Xavier D. E. Costa W. K. A. Oliveira K. Franco O. L. (2016). Identification of lactic acid bacteria in fruit pulp processing byproducts and potential probiotic properties of selected *Lactobacillus* strains. *Front. Microbiol.* 7:1371. 10.3389/fmicb.2016.01371 27625647 PMC5003889

[B23] García-Hernández J. Hernández-Pérez M. Peinado I. Andrés A. Heredia A. (2018). Tomato-antioxidants enhance viability of *L. reuteri* under gastrointestinal conditions while the probiotic negatively affects bioaccessibility of lycopene and phenols. *J. Funct. Foods* 43 1–7. 10.1016/j.jff.2017.12.052

[B24] Gelen V. şengül E. Gedikli S. Gür C. Özkanlar S. (2017). Therapeutic effect of quercetin on renal function and tissue damage in the obesity induced rats. *Biomed. Pharmacother.* 89 524–528. 10.1016/j.biopha.2017.02.057 28254664

[B25] Georgakouli K. Mpesios A. Kouretas D. Petrotos K. Mitsagga C. Giavasis I. (2016). The effects of an olive fruit polyphenol-enriched yogurt on body composition, blood redox status, physiological and metabolic parameters and yogurt microflora. *Nutrients* 8 344. 10.3390/nu8060344 27271664 PMC4924185

[B26] Harwood M. Danielewska-Nikiel B. Borzelleca J. F. Flamm G. W. Williams G. M. Lines T. C. (2007). A critical review of the data related to the safety of quercetin and lack of evidence of *in vivo* toxicity, including lack of genotoxic/carcinogenic properties. *Food Chem. Toxicol.* 45 2179–2205. 10.1016/j.fct.2007.05.015 17698276

[B27] Hervert-Hernández D. Pintado C. Rotger R. Goñi I. (2009). Stimulatory role of grape pomace polyphenols on *Lactobacillus acidophilus* growth. *Int. J. Food. Microbiol.* 136 119–122. 10.1016/j.ijfoodmicro.2009.09.016 19836092

[B28] Hossen M. S. Ali M. Y. Jahurul M. H. A. Abdel-Daim M. M. Gan S. H. Khalil M. I. (2017). Beneficial roles of honey polyphenols against some human degenerative diseases: a review. *Pharmacol. Rep.* 69 1194–1205. 10.1016/j.pharep.2017.07.002 29128800

[B29] Jacobsen C. N. Nielsen V. R. Hayford A. E. Møller P. L. Michaelsen K. F. Paerregaard A. (1999). Screening of probiotic activities of forty-seven strains of *Lactobacillus* spp. *by in vitro* techniques and evaluation of the colonization ability of five selected strains in humans. *Appl. Environ. Microbiol.* 65 4949–4956. 10543808 10.1128/aem.65.11.4949-4956.1999 PMC91666

[B30] Karam J. Bibiloni M. M. Tur J. A. (2018). Polyphenol estimated intake and dietary sources among older adults from Mallorca Island. *PLoS One* 13:e0191573. 10.1371/journal.pone.0191573 29381732 PMC5790249

[B31] Khalil R. K. (2010). Influence of gallic acid and catechin polyphenols on probiotic properties of *Streptococcus thermophilus* CHCC 3534 strain. *World J. Microbiol. Biotechnol.* 26 2069–2079. 10.1007/s11274-010-0393-8

[B32] Kiely L. J. Olson N. F. (2000). The physicochemical surface characteristics of *Lactobacillus casei*. *Food Microbiol.* 17 277–291. 10.1006/fmic.1999.0311

[B33] Lacey A. M. L. Pérez-Santín E. López-Caballero M. E. Montero P. (2014). Biotransformation and resulting biological properties of green tea polyphenols produced by probiotic bacteria. *LWT Food Sci. Technol.* 58 633–638. 10.1016/j.lwt.2014.03.040

[B34] Lebeer S. Vanderleyden J. De Keersmaecker S. C. (2008). Genes and molecules of lactobacilli supporting probiotic action. *Microbiol. Mol. Biol. Rev.* 72 728–764. 10.1128/MMBR.00017-08 19052326 PMC2593565

[B35] Llano D. G. Gil-Sánchez I. Esteban-Fernández A. Ramos A. M. Fernández-Díaz M. Cueva C. (2017). Reciprocal beneficial effects between wine polyphenols and probiotics: an exploratory study. *Eur. Food Res. Technol.* 243 531–538. 10.1007/s00217-016-2770-5

[B36] Maukonen J. Saarela M. (2015). Human gut microbiota: does diet matter? *Proc. Nutr. Soc.* 74 23–36. 10.1017/S0029665114000688 25156389

[B37] Monteagudo-Mera A. Rodrıguez-Aparicio L. Rua J. Martinez B. H. Navasa N. Garcia-Armesto R. M. (2012). *In vitro* evaluation of physiological probiotic properties of different lactic acid bacteria strains of dairy and human origin. *J. Funct. Foods* 4 531–541. 10.1016/j.jff.2012.02.014

[B38] Mora-Pale M. Bhan N. Masuko S. James P. Wood J. McCallum S. (2015). Antimicrobial mechanism of resveratrol-trans-dihydrodimer produced from peroxidase-catalyzed oxidation of resveratrol. *Biotechnol. Bioeng.* 112 2417–2428. 10.1002/bit.25686 26109045

[B39] Moreno-Indias I. Sánchez-Alcoholado L. Pérez-Martínez P. Andrés-Lacueva C. Cardona F. Tinahones F. (2016). Red wine polyphenols modulate fecal microbiota and reduce markers of the metabolic syndrome in obese patients. *Food Funct.* 7 1775–1787. 10.1039/C5FO00886 26599039

[B40] Mueller M. Zartl B. Schleritzko A. Stenzl M. Viernstein H. Unger F. M. (2018). Rhamnosidase activity of selected probiotics and their ability to hydrolyse flavonoid rhamnoglucosides. *Bioprocess Biosyst. Eng.* 41 221–228. 10.1007/s00449-017-1860-5 29124335 PMC5773629

[B41] Pacheco-Ordaz R. Wall-Medrano A. Goñi M. G. Ramos-Clamont-Montfort G. Ayala-Zavala J. F. González-Aguilar G. A. (2018). Effect of phenolic compounds on the growth of selected probiotic and pathogenic bacteria. *Lett. Appl. Microbiol.* 66 25–31. 10.1111/lam.12814 29063625

[B42] Sergides C. Chirila M. Silvestro L. Pitta D. Pittas A. (2016). Bioavailability and safety study of resveratrol 500 mg tablets in healthy male and female volunteers. *Exp. Ther. Med.* 11 164–170. 10.3892/etm.2015.2895 26889234 PMC4726856

[B43] Su P. Henriksson A. Nilsson C. Mitchell H. (2008). Synergistic effect of green tea extract and probiotics on the pathogenic bacteria, *Staphylococcus aureus* and *Streptococcus pyogenes*. *World J. Microbiol. Biotechnol.* 24 1837–1842. 10.1007/s11274-008-9682-x

[B44] Tabasco R. Sánchez-Patán F. Monagas M. Bartolomé B. Moreno-Arribas M. V. Peláez C. (2011). Effect of grape polyphenols on lactic acid bacteria and bifidobacteria growth: resistance and metabolism. *Food Microbiol.* 28 1345–1352. 10.1016/j.fm.2011.06.005 21839384

[B45] Taguchi C. Fukushima Y. Kishimoto Y. Suzuki-Sugihara N. Saita E. Takahashi Y. (2015). Estimated dietary polyphenol intake and major food and beverage sources among elderly japanese. *Nutrients* 7 10269–10281. 10.3390/nu7125530 26690212 PMC4690082

[B46] Todorov S. D. Botes M. Guigas C. Schillinger U. Wiid I. Wachsman M. B. (2008). Boza, a natural source of probiotic lactic acid bacteria. *J. Appl. Microbiol.* 104 465–477. 10.1111/j.1365-2672.2007.03558.x 17922827

[B47] Tome-Carneiro J. Larrosa M. Gonzalez-Sarrias A. Tomas-Barberan F. A. Garcia-Conesa M. T. Espin J. C. (2013). Resveratrol and clinical trials: the crossroad from *in vitro* studies to human evidence. *Curr. Pharm. Des.* 19 6064–6093. 10.2174/13816128113199990407 23448440 PMC3782695

[B48] van Vuuren S. F. (2008). Antimicrobial activity of South African medicinal plants. *J. Ethnopharmacol.* 119 462–472. 10.1016/j.jep.2008.05.038 18582553

[B49] Ventura M. O’Flaherty S. Claesson M. J. Turroni F. Klaenhammer T. R. van Sinderen D. (2009). Genome-scale analyses of health promoting bacteria: probiogenomics. *Nat. Rev. Microbiol.* 7 61–71. 10.1038/nrmicro2047 19029955

[B50] Volstatova T. Marsik P. Rada V. Geigerova M. Havlik J. (2017). Effect of apple extracts and selective polyphenols on the adhesion of potential probiotic strains of *Lactobacillus gasseri* R and *Lactobacillus casei* FMP. *J. Funct. Foods* 35 391–397. 10.1016/j.jff.2017.06.005

[B51] Wang S. Yao J. Zhou B. Yang J. Chaudry M. T. Wang M. (2017). Bacteriostatic effect of quercetin as an antibiotic alternative *in vivo* and its antibacterial mechanism *in vitro*. *J. Food. Prot.* 81 68–78. 10.4315/0362-028X.JFP-17-214 29271686

