# Supplementary material for: Effects of Quercetin and Resveratrol on in vitro Properties Related to the Functionality of Potentially Probiotic Lactobacillus Strains
Source: Front Microbiol. 2019 Sep 24;10:2229. doi: 10.3389/fmicb.2019.02229 (PMC6769296; doi:10.3389/fmicb.2019.02229)
Supplement: Supplementary file 1 [file Table_1.DOCX]

Supplementary Material

| Table S1. Viable counts (n = 9; average ± standard deviation; log CFU/mL) of potentially probiotic *Lactobacillus* strains when exposed to pH 7.2 and 5 in media with and without different concentrations of quercetin (QUE) or resveratrol (RES) for different time intervals. | | | | | | |
| --- | --- | --- | --- | --- | --- | --- |
| Strains/treatments | pH values/exposure times | | |  | | |
|  | pH 7.2 | | | pH 5 | | |
|  | 1 h | 2 h | 3 h | 1 h | 2 h | 3 h |
| *L. plantarum* 49 |  |  |  |  |  |  |
| MIC QUE | 7.4 ± 0.6 | 7.5 ± 0.6 | 7.4 ± 0.5 | 7.3 ± 0.6 | 7.4 ± 0.4 | 7.5 ± 0.5 |
| 1/2 MIC QUE | 7.4 ± 0.5 | 7.7 ± 0.9 | 6.8 ± 0.4 | 7.2 ± 0.3 | 6.7 ± 0.4 | 6.4 ± 0.5 |
| 1/4 MIC QUE | 7.2 ± 0.3 | 7.7 ± 0.8 | 6.5 ± 0.6 | 7.6 ± 0.2 | 7.3 ± 0.5 | 7.3 ± 0.2 |
| MIC RES | 7.4 ± 0.2 | 7.4 ± 0.5 | 7.4 ± 0.3 | 7.5 ± 0.2 | 7.4 ± 0.5 | 7.2 ± 0.2 |
| 1/2 MIC RES | 7.2 ± 0.2 | 6.6 ± 0.6 | 7.4 ± 0.4 | 7.1 ± 0.2 | 7.2 ± 0.5 | 7.2 ± 0.3 |
| 1/4 MIC RES | 6.8 ± 0.2 | 6.7 ± 0.5 | 6.6 ± 0.5 | 7.4 ± 0.2 | 7.3 ± 0.2 | 7.3 ± 0.4 |
| Control | 7.3 ± 0.7 | 7.6 ± 0.5 | 7.4 ± 0.3 | 7.5 ± 0.2 | 7.1 ± 0.3 | 7.4 ± 0.3 |
| *L. plantarum* 53 |  |  |  |  |  |  |
| MIC QUE | 7.2 ± 0.6 | 6.6 ± 0.2 | 6.3 ± 0.2 | 6.2 ± 0.8 | 7.6 ± 0.4^A^ | 5.9 ± 0.5 |
| 1/2 MIC QUE | 7.3 ± 0.2 | 6.7 ± 0.3 | 6.4 ± 0.2 | 6.4 ± 0.6 | 6.6 ± 0.3^B^ | 6.2 ± 0.2 |
| 1/4 MIC QUE | 7.0 ± 0.3 | 6.5 ± 0.7 | 6.5 ± 0.4 | 6.6 ± 0.3 | 6.4 ± 0.5^B^ | 6.1 ± 0.2 |
| MIC RES | 7.3 ± 0.4 | 6.7 ± 0.2 | 6.6 ± 0.2 | 7.3 ± 0.6 | 6.6 ± 0.3^B^ | 5.9 ± 0.2 |
| 1/2 MIC RES | 6.8 ± 0.5 | 6.4 ± 0.6 | 6.3 ± 0.2 | 6.6 ± 0.5^A^ | 6.7 ± 0.2^B^ | 6.2 ± 0.2 |
| 1/4 MIC RES | 6.6 ± 0.4 | 6.7 ± 0.5 | 6.5 ± 0.3 | 6.8 ± 0.3^A^ | 6.7 ± 0.3^B^ | 5.7 ± 0.3 |
| Control | 7.4 ± 0.5 | 6.8 ± 0.3 | 6.5 ± 0.5 | 6.8 ± 0.2^A^ | 7.5 ± 0.2^A^ | 6.4 ± 0.4 |
| *L. paracasei* 106 |  |  |  |  |  |  |
| MIC QUE | 6.6 ± 0.3 | 6.5 ± 0.7^AB^ | 6.7 ± 0.5 | 6.4 ± 0.8 | 6.2 ± 0.2 | 6.1 ± 0.7^A^ |
| 1/2 MIC QUE | 6.7 ± 0.4 | 6.2 ± 0.3^B^ | 6.4 ± 0.6 | 6.5 ± 0.4 | 6.3 ± 0.3 | 4.4 ± 0.5^B^ |
| 1/4 MIC QUE | 6.3 ± 0.2 | 6.8 ± 0.3^AB^ | 6.6 ± 0.6 | 6.3 ± 0.7 | 5.9 ± 0.4 | 5.4 ± 0.7^AB^ |
| MIC RES | 6.1 ± 0.6 | 6.7 ± 0.3^AB^ | 6.8 ± 0.4 | 6.3 ± 0.4 | 6.1 ± 0.3 | 5.2 ± 0.4^B^ |
| 1/2 MIC RES | 6.7 ± 0.6 | 6.5 ± 0.7^AB^ | 6.1 ± 0.7 | 6.2 ± 0.2 | 6.1 ± 0.6 | 4.6 ± 0.6^B^ |
| 1/4 MIC RES | 6.3 ± 0.3 | 6.5 ± 0.6^AB^ | 6.7 ± 0.5 | 6.4 ± 0.4 | 6.4 ± 0.8 | 4.2 ± 0.7^B^ |
| Control | 6.6 ± 0.5 | 7.1 ± 0.5 ^A^ | 6.7 ± 0.3 | 6.4 ± 0.3 | 6.3 ± 0.3 | 6.3 ± 0.4^A^ |
| *L. paracasei* 108 |  |  |  |  |  |  |
| MIC QUE | 7.2 ± 0.5 | 5.2 ± 0.3^B^ | 5.2 ± 0.4^B^ | 4.1 ± 0.8^B^ | 5.5 ± 0.5^B^ | 4.8 ± 1.1^AB^ |
| 1/2 MIC QUE | 7.2 ± 0.8 | 6.6 ± 0.3^A^ | 5.9 ± 1.3^AB^ | 5.7 ± 0.7^AB^ | 4.8 ± 0.4^BC^ | 4.9 ± 1.3^AB^ |
| 1/4 MIC QUE | 7.5 ± 0.7 | 6.5 ± 0.6^AB^ | 6.6 ± 0.4^A^ | 5.6 ± 0.7^AB^ | 6.6 ± 0.9^AB^ | 4.4 ± 0.6^AB^ |
| MIC RES | 6.4 ± 0.6 | 5.0 ± 0.6^AB^ | 6.5 ± 0.7^A^ | 5.9 ± 0.4^A^ | 7.3 ± 0.5^A^ | 5.6 ± 0.6^A^ |
| 1/2 MIC RES | 6.7 ± 0.4 | 5.4 ± 0.3^AB^ | 5.8 ± 1.0^AB^ | 6.2 ± 0.9^A^ | 4.3 ± 0.9^BC^ | 4.4 ±0.4^B^ |
| 1/4 MIC RES | 6.8 ± 0.3 | 5.5 ± 0.2^AB^ | 5.5 ± 0.7^AB^ | 6.3 ± 1.1^A^ | 4.2 ± 0.4^C^ | 4.6 ±0.5^AB^ |
| Control | 6.3 ± 0.5 | 5.8 ± 1.3^A^ | 5.9 ± 0.9^AB^ | 6.3 ± 0.4^A^ | 6.6 ± 0.2^A^ | 5.4 ± 0.7^AB^ |
| *L. fermentum* 263 |  |  |  |  |  |  |
| MIC QUE | 6.8 ± 0.9 | 7.5 ± 0.7^A^ | 5.6 ± 0.2^B^ | 7.5 ± 0.9^AB^ | 5.2 ± 0.2^B^ | 5.5 ± 0.4^B^ |
| 1/2 MIC QUE | 6.2 ± 0.8 | 6.9 ± 0.4^A^ | 5.4 ± 0.6^B^ | 7.3 ± 0.6^AB^ | 6.4 ± 0.5^A^ | 5.8 ± 0.6^AB^ |
| 1/4 MIC QUE | 7.1 ± 1.0 | 7.4 ± 0.3^A^ | 6.2 ± 0.5^B^ | 7.6 ± 0.7^AB^ | 6.5 ± 0.4^A^ | 5.0 ± 0.6^B^ |
| MIC RES | 6.9 ± 0.2 | 6.4 ± 0.7^B^ | 5.6 ± 0.4^B^ | 7.0 ± 0.4^AB^ | 5.3 ± 0.4^B^ | 4.7 ± 0.5^B^ |
| 1/2 MIC RES | 7.4 ± 0.5 | 6.6 ± 0.4^B^ | 5.9 ± 0.7^B^ | 6.1 ± 0.8^B^ | 6.9 ± 0.2^A^ | 5.9 ± 0.7^AB^ |
| 1/4 MIC RES | 7.5 ± 0.7 | 7.6 ± 0.6^A^ | 6.5 ± 0.5^AB^ | 7.3 ± 0.3^AB^ | 7.4 ± 0.6^A^ | 6.3 ± 0.2^A^ |
| Control | 7.4 ± 0.3 | 7.5 ± 0.5^A^ | 7.5 ± 0.5^A^ | 7.6 ± 0.3^A^ | 7.3 ± 0.5^A^ | 6.6 ± 0.3^A^ |
| *L. fermentum* 296 |  |  |  |  |  |  |
| MIC QUE | 6.5 ± 0.4^B^ | 6.4 ± 0.5 | 4.3 ± 1.1^B^ | 7.4 ± 0.3^A^ | 5.8 ± 2.3 | 7.7 ± 0.5 |
| 1/2 MIC QUE | 7.7 ± 0.3^A^ | 6.2 ± 0.4 | 5.5 ± 0.5^AB^ | 6.3 ± 0.4^B^ | 6.6 ± 0.3 | 6.9 ± 1.6 |
| 1/4 MIC QUE | 6.1 ± 0.2^B^ | 6.5 ± 0.7 | 5.6 ± 0.2^B^ | 7.2 ± 0.3^A^ | 5.9 ± 2.0 | 5.7 ± 1.8 |
| MIC RES | 6.5 ± 0.9^AB^ | 6.5 ± 0.6 | 6.2 ± 0.5^A^ | 6.5 ± 0.2^B^ | 6.3 ± 1.2 | 7.7 ± 0.4 |
| 1/2 MIC RES | 6.6 ± 0.4^B^ | 5.9 ± 0.9 | 6.6 ± 0.6^A^ | 6.5 ± 0.6^AB^ | 6.3 ± 0.8 | 6.9 ±1.5 |
| 1/4 MIC RES | 6.5 ± 0.8^B^ | 6.0 ± 0.5 | 6.5 ± 0.5^A^ | 5.3 ± 0.5^C^ | 6.5 ± 0.4 | 7.7 ± 0.5 |
| Control | 6.4 ± 0.4^B^ | 5.4 ± 0.5 | 4.4 ± 0.5^B^ | 7.4 ± 0.6^A^ | 6.6 ± 0.3 | 6.9 ± 1.6 |
| Control: 0 µg/mL of QUE or RES.  For *L. plantarum* 49, *L. plantarum* 53 and *L. fermentum* 263 - MIC of QUE: 1024 µg/mL, 1/2 MIC of QUE: 512 µg/mL, 1/4 MIC of QUE: 256 µg/mL, MIC of RES: 1024 µg/mL, 1/2 MIC of RES: 512 µg/mL, 1/4 MIC of RES: 256 µg/mL; For *L. paracasei* 108 and *L. fermentum* 296 - MIC of QUE: 512 µg/mL, 1/2 MIC of QUE: 256 µg/mL, 1/4 MIC of QUE: 128 µg/mL, MIC of RES: 1024 µg/mL, 1/2 MIC of RES: 512 µg/mL, 1/4 MIC of RES: 256 µg/mL; For *L. paracasei* 106 - MIC of QUE: 1024 µg/mL, 1/2 MIC of QUE: 512 µg/mL, 1/4 MIC of QUE: 256 µg/mL, MIC of RES: 1024 µg/mL (assumed), 1/2 MIC of RES: 512 µg/mL (assumed), 1/4 MIC of RES: 256 µg/mL (assumed).  ^A – C^: Different superscript capital letters in the same column, for the same *Lactobacillus* strain, denote difference (p ≤ 0.05) among the counts when exposed to a pH for a specific time interval in media with or without different concentrations of QUE or RES, based on Tukey’s test. The absence of superscript capital letters denotes no difference (p > 0.05) among these counts. | | | | | | |

| Table S2. Viable counts (n = 9; average ± standard deviation; log CFU/mL) of potentially probiotic *Lactobacillus* strains when exposed to different bile salt concentrations (w/v) in media with and without different amounts of quercetin (QUE) or resveratrol (RES) for different time intervals. | | | | | | | | | | | | |
| --- | --- | --- | --- | --- | --- | --- | --- | --- | --- | --- | --- | --- |
| Treatments | Bile salts concentrations/exposure time | | |  | | |  | | |  | | |
|  | 0% | | | 0.15% | | | 0.3% | | | 1% | | |
|  | 1 h | 2 h | 3 h | 1 h | 2 h | 3 h | 1 h | 2 h | 3 h | 1 h | 2 h | 3 h |
| *L. plantarum* 49 |  | | | | | | | | | | | |
| MIC QUE | 5.9 ± 0.8^AB^ | 5.1 ± 0.5^B^ | 5.1 ± 0.8 | 4.9 ± 0.8^AB^ | 4.4 ± 0.8 | 4.2 ± 0.2^B^ | 6.0 ± 0.7 | 5.5 ± 0.4 | 5.4 ± 0.3 | 6.2 ± 0.2 | 6.0 ± 0.9 | 5.0 ± 0.3^B^ |
| 1/2 MIC QUE | 5.4 ± 0.9^AB^ | 5.1 ± 0.5^B^ | 5.7 ± 0.6 | 5.7 ± 0.3^A^ | 4.4 ± 0.3 | 4.4 ± 0.2^B^ | 6.1 ± 0.7 | 5.4 ± 0.7 | 5.0 ± 0.8 | 6.1 ± 0.2 | 6.5 ± 0.7 | 5.9 ± 0.3^A^ |
| 1/4 MIC QUE | 6.3 ± 0.2^AB^ | 5.8 ± 0.7^AB^ | 5.8 ± 0.6 | 4.8 ± 0.4^B^ | 4.9 ± 0.4 | 5.6 ± 0.4^A^ | 5.9 ± 0.7 | 5.5 ± 0.5 | 5.4 ± 0.3 | 6.0 ± 0.5 | 6.0 ± 0.9 | 5.9 ± 0.2^A^ |
| MIC RES | 6.0 ± 0.3^AB^ | 5.6 ± 1.9^AB^ | 5.6 ± 0.3 | 5.1 ± 0.2^B^ | 4.9 ± 0.3 | 4.7 ± 0.2^B^ | 5.9 ± 0.8 | 5.5 ± 0.9 | 5.5 ± 0.5 | 6.2 ± 0.8 | 6.1 ± 0.2 | 5.9 ± 0.3^A^ |
| 1/2 MIC RES | 5.7 ± 0.4^B^ | 5.5 ± 0.7^B^ | 5.4 ± 0.5 | 5.4 ± 0.4^AB^ | 4.9 ± 0.3 | 4.7 ± 0.6^AB^ | 6.0 ± 0.8 | 5.5 ± 0.6 | 5.4 ± 0.4 | 6.0 ± 0.3 | 6.2 ± 0.4 | 5.7 ± 0.5^AB^ |
| 1/4 MIC RES | 6.7 ± 0.5^A^ | 7.1 ± 0.7^A^ | 5.8 ± 0.2 | 5.3 ± 0.3^AB^ | 5.1 ± 0.4 | 4.8 ± 0.5^AB^ | 4.9 ± 0.8 | 5.5 ± 0.8 | 5.4 ± 0.2 | 5.5 ± 0.7 | 5.3 ± 0.7 | 5.9 ± 0.2^A^ |
| Control | 6.6 ± 0.4^A^ | 6.3 ± 0.7^AB^ | 5.5 ± 0.2 | 5.9 ± 0.4^A^ | 4.8 ± 0.2 | 4.6 ± 0.4^B^ | 5.8 ± 0.8 | 5.5 ± 0.3 | 5.5 ± 0.5 | 6.1 ± 0.4 | 6.1 ± 0.5 | 5.0 ± 0.2^B^ |
| *L. plantarum* 53 |  | | | | | | | | | | | |
| MIC QUE | 7.1 ± 0.4 | 7.1 ± 0.5^A^ | 6.5 ± 0.9^A^ | 5.9 ± 0.6 | 4.8 ± 0.9 | 4.8 ± 0.5 | 6.9 ± 0.8 | 5.9 ± 0.5^AB^ | 5.4 ± 0.9 | 5.6 ± 0.9^AB^ | 5.1 ± 0.5 | 4.9 ± 0.6 |
| 1/2 MIC QUE | 6.7 ± 0.5 | 5.9 ± 0.4^B^ | 5.5 ± 0.8^B^ | 5.6 ± 0.9 | 5.1 ± 0.4 | 4.9 ± 0.8 | 6.3 ± 0.2 | 5.4 ± 0.3^B^ | 4.9 ± 0.3 | 5.6 ± 0.4^A^ | 5.7 ± 0.5 | 4.8 ± 0.7 |
| 1/4 MIC QUE | 7.1 ± 0.5 | 6.7 ± 0.5^AB^ | 5.9 ± 0.3^B^ | 6.1 ± 0.5 | 5.1 ± 0.7 | 4.8 ± 0.5 | 6.6 ± 0.5 | 5.7 ± 0.5^AB^ | 5.3 ± 0.9 | 5.6 ± 0.6^A^ | 5.9 ± 0.9 | 5.7 ± 0.4 |
| MIC RES | 6.4 ± 0.6 | 5.6 ± 0.6^B^ | 4.8 ± 0.8^B^ | 5.7 ± 0.3 | 5.2 ± 0.4 | 4.7 ± 0.2 | 6.9 ± 0.8 | 6.8 ± 0.6^A^ | 5.7 ± 0.7 | 5.2 ± 0.3^AB^ | 5.8 ± 0.5 | 5.6 ± 0.2 |
| 1/2 MIC RES | 6.5 ± 0.4 | 5.5 ± 0.7^B^ | 5.5 ± 0.2^B^ | 5.5 ± 0.4 | 4.9 ± 0.8 | 4.9 ± 1.2 | 6.8 ± 0.6 | 6.3 ± 0.2^A^ | 5.7 ± 0.8 | 5.2 ± 0.5^AB^ | 5.6 ± 0.2 | 5.2 ± 0.3 |
| 1/4 MIC RES | 7.5 ± 0.6 | 6.5 ± 0.7^AB^ | 6.0 ± 0.3^B^ | 6.2 ± 0.4 | 4.8 ± 1.2 | 4.8 ± 1.0 | 6.8 ± 0.4 | 6.1 ± 0.6^AB^ | 5.8 ± 0.6 | 4.2 ± 0.7^B^ | 5.6 ± 0.5 | 5.2 ± 0.2 |
| Control | 7.2 ± 0.3 | 7.7 ± 0.6^A^ | 6.9 ± 0.3^A^ | 5.9 ± 0.3 | 5.4 ± 0.5 | 5.4 ± 1.1 | 6.6 ± 0.9 | 6.1 ± 0.5^AB^ | 5.3 ± 0.6 | 5.0 ± 1.0^AB^ | 5.7 ± 0.4 | 5.3 ± 0.4 |
| *L. paracasei* 106 |  | | | | | | | | | | | |
| MIC QUE | 6.0 ± 0.9^AB^ | 5.1 ± 1.0^B^ | 5.3 ± 0.5^A^ | 6.6 ± 0.3 | 6.1 ± 0.5 | 5.7 ± 0.4 | 6.1 ± 0.5 | 5.2 ± 0.8 | 5.4 ± 0.7 | 4.3 ± 0.6 | 5.3 ± 0.8 | 4.4 ± 0.8 |
| 1/2 MIC QUE | 6.3 ± 0.5^AB^ | 5.3 ± 0.8^B^ | 5.6 ± 0.5^A^ | 5.9 ± 0.6 | 6.1 ± 0.3 | 6.3 ± 0.5 | 6.3 ± 0.5 | 5.3 ± 1.0 | 5.9 ± 0.2 | 4.0 ± 0.4 | 5.2 ± 1.2 | 4.3 ± 0.9 |
| 1/4 MIC QUE | 7.3 ± 0.5^A^ | 6.1 ± 0.6^AB^ | 7.1 ± 1.4^A^ | 6.5 ± 0.3 | 6.2 ± 0.4 | 5.9 ± 0.3 | 6.4 ± 0.2 | 5.2 ± 1.3 | 5.7 ± 0.2 | 3.9 ± 0.9 | 4.8 ± 1.1 | 4.9 ± 1.3 |
| MIC RES | 7.2 ± 0.5^A^ | 5.7 ± 1.0^AB^ | 6.2 ± 0.4^A^ | 6.6 ± 0.5 | 6.8 ± 0.4 | 5.4 ± 0.5 | 6.6 ± 0.5 | 5.5 ± 1.7 | 5.2 ± 1.0 | 4.0 ± 0.4 | 4.4 ± 0.2 | 5.3 ± 0.6 |
| 1/2 MIC RES | 5.5 ± 0.5^B^ | 5.6 ± 0.5^B^ | 5.0 ± 0.4^B^ | 6.6 ± 0.5 | 6.3 ± 0.5 | 5.9 ± 0.9 | 6.6 ± 0.8 | 5.5 ± 1.4 | 5.8 ± 0.2 | 4.1 ± 0.3 | 4.9 ± 0.9 | 5.1 ± 0.5 |
| 1/4 MIC RES | 7.3 ± 0.5^A^ | 7.1 ± 0.6^A^ | 6.4 ± 0.6^A^ | 6.6 ± 0.2 | 6.3 ± 0.4 | 5.7 ± 0.5 | 6.5 ± 0.4 | 5.5 ± 1.6 | 5.4 ± 0.6 | 4.0 ± 0.4 | 4.7 ± 0.5 | 5.0 ± 0.5 |
| Control | 6.4 ± 1.5^AB^ | 6.5 ± 0.9^AB^ | 6.5 ± 0.7^A^ | 6.3 ± 1.2 | 6.2 ± 0.2 | 5.7 ± 0.9 | 6.3 ± 0.8 | 6.0 ± 0.4 | 5.7 ± 0.5 | 4.3 ± 0.7 | 5.3 ± 0.8 | 4.9 ± 1.2 |
| *L. paracasei* 108 |  | | | | | | | | | | | |
| MIC QUE | 7.1 ± 0.4 | 6.4 ± 0.6 | 6.6 ± 0.2 | 4.8 ± 0.7 | 4.5 ± 0.5 | 4.0 ± 0.6 | 3.5 ± 0.6^B^ | < 1 ± 0.0 | < 1 ± 0.0 | < 1 ± 0.0 | < 1 ± 0.0 | < 1 ± 0.0 |
| 1/2 MIC QUE | 7.1 ± 0.6 | 7.1 ± 0.7 | 6.8 ± 0.7 | 4.8 ± 0.7 | 4.3 ± 0.6 | 4.3 ± 0.6 | 4.6 ± 0.5^A^ | < 1 ± 0.0 | < 1 ± 0.0 | < 1 ± 0.0 | < 1 ± 0.0 | < 1 ± 0.0 |
| 1/4 MIC QUE | 7.1 ± 0.5 | 6.9 ± 0.8 | 6.9 ± 0.9 | 4.6 ± 0.5 | 4.7 ± 1.4 | 4.3 ± 0.8 | 4.6 ± 0.2^A^ | < 1 ± 0.0 | < 1 ± 0.0 | < 1 ± 0.0 | < 1 ± 0.0 | < 1 ± 0.0 |
| MIC RES | 7.2 ± 0.8 | 7.1 ± 0.7 | 7.1 ± 0.4 | 4.9 ± 0.6 | 4.2 ± 1.1 | 3.4 ± 1.5 | 3.8 ± 0.2^B^ | < 1 ± 0.0 | < 1 ± 0.0 | < 1 ± 0.0 | < 1 ± 0.0 | < 1 ± 0.0 |
| 1/2 MIC RES | 6.3 ± 0.4 | 6.1 ± 0.2 | 6.1 ± 0.6 | 4.4 ± 0.7 | 3.9 ± 0.6 | 3.4 ± 0.9 | 3.7 ± 0.3^B^ | < 1 ± 0.0 | < 1 ± 0.0 | < 1 ± 0.0 | < 1 ± 0.0 | < 1 ± 0.0 |
| 1/4 MIC RES | 6.8 ± 0.7 | 6.8 ± 0.9 | 6.8 ± 0.8 | 5.1 ± 0.7 | 4.8 ± 1.2 | 4.5 ± 0.4 | 2.9 ± 0.3^B^ | < 1 ± 0.0 | < 1 ± 0.0 | < 1 ± 0.0 | < 1 ± 0.0 | < 1 ± 0.0 |
| Control | 6.9 ± 0.6 | 6.8 ± 1.0 | 7.0 ± 0.7 | 5.3 ± 0.6 | 5.7 ± 1.4 | 4.9 ± 0.7 | 4.9 ± 0.2^A^ | < 1 ± 0.0 | < 1 ± 0.0 | < 1 ± 0.0 | < 1 ± 0.0 | < 1 ± 0.0 |
| *L. fermentum* 263 |  | | | | | | | | | | | |
| MIC QUE | 5.9 ± 1.2^AB^ | 6.5 ± 0.7^AB^ | 5.6 ± 0.7^B^ | 5.5 ± 0.5 | 5.6 ± 0.8 | 5.5 ± 0.7 | 7.3 ± 0.6 | 7.3 ± 0.4 | 7.2 ± 0.5 | 7.0 ± 0.5 | 6.3 ± 0.5 | 5.4 ± 0.6 |
| 1/2 MIC QUE | 6.6 ± 0.6^AB^ | 6.1 ± 0.6^B^ | 5.7 ± 0.9^AB^ | 5.6 ± 0.5 | 6.3 ± 0.6 | 5.6 ± 1.0 | 7.2 ± 0.5 | 7.2 ± 0.2 | 6.9 ± 0.5 | 6.9 ± 0.5 | 6.5 ± 0.4 | 5.9 ± 0.7 |
| 1/4 MIC QUE | 6.9 ± 0.4^A^ | 6.8 ± 0.5^AB^ | 6.0 ± 0.6^AB^ | 5.5 ± 0.7 | 5.6 ± 0.6 | 5.6 ± 0.8 | 7.2 ± 0.9 | 7.3 ± 0.3 | 7.3 ± 0.6 | 7.0 ± 0.6 | 6.4 ± 0.5 | 6.0 ± 0.6 |
| MIC RES | 6.9 ± 0.5^AB^ | 6.7 ± 0.4^AB^ | 6.1 ± 0.5^AB^ | 5.6 ± 0.6 | 5.6 ± 0.5 | 6.4 ± 0.6 | 7.4 ± 0.7 | 7.1 ± 0.2 | 6.9 ± 0.5 | 7.1 ± 0.4 | 6.2 ± 0.6 | 4.9 ± 0.5 |
| 1/2 MIC RES | 5.7 ± 0.7^B^ | 5.9 ± 0.5^B^ | 6.6 ± 0.6^AB^ | 5.6 ± 0.5 | 5.5 ± 0.7 | 5.6 ± 0.9 | 7.4 ± 0.6 | 7.2 ± 0.3 | 7.1 ± 0.6 | 7.1 ± 0.6 | 6.3 ± 0.6 | 5.1 ± 0.6 |
| 1/4 MIC RES | 7.1 ± 0.9^AB^ | 7.1 ± 0.3^A^ | 7.1 ± 0.5^A^ | 5.6 ± 0.5 | 5.6 ± 0.6 | 5.6 ± 0.6 | 7.4 ± 0.5 | 7.0 ± 0.4 | 7.0 ± 0.5 | 7.1 ± 0.5 | 6.1 ± 0.6 | 4.9 ± 0.5 |
| Control | 7.4 ± 0.4^A^ | 7.3 ± 0.2^A^ | 7.0 ± 0.5^A^ | 5.9 ± 1.0 | 5.8 ± 0.6 | 5.7 ± 0.5 | 7.0 ± 0.7 | 6.9 ± 0.2 | 6.7 ± 0.5 | 6.7 ± 0.6 | 6.3 ± 0.7 | 4.9 ± 0.6 |
| *L. fermentum* 296 |  | | | | | | | | | | | |
| MIC QUE | 6.4 ± 0.6^AB^ | 6.1 ± 0.7^AB^ | 5.7 ± 0.4^B^ | 5.8 ± 0.6 | 5.7 ± 0.4 | 5.4 ± 0.5 | 7.3 ± 0.6 | 7.0 ± 0.6 | 6.7 ± 0.3 | 7.1 ± 0.7 | 6.1 ± 0.7 | 5.3 ± 0.6 |
| 1/2 MIC QUE | 5.7 ± 0.4^B^ | 5.9 ± 0.5^B^ | 5.7 ± 0.5^B^ | 6.6 ± 0.6 | 5.8 ± 0.6 | 5.5 ± 0.6 | 7.4 ± 0.6 | 7.3 ± 0.6 | 6.7 ± 0.4 | 7.2 ± 0.6 | 6.3 ± 0.6 | 5.4 ± 0.5 |
| 1/4 MIC QUE | 5.7 ± 0.5^B^ | 6.1 ± 0.6^B^ | 6.5 ± 0.7^AB^ | 6.5 ± 0.7 | 5.5 ± 0.9 | 5.3 ± 0.6 | 7.3 ± 0.6 | 7.4 ± 0.6 | 7.1 ± 0.6 | 7.1 ± 0.5 | 5.9 ± 0.5 | 5.3 ± 1.6 |
| MIC RES | 5.6 ± 0.6^B^ | 5.1 ± 0.5^B^ | 5.3 ± 0.6^B^ | 6.5 ± 0.7 | 5.7 ± 0.5 | 4.9 ± 0.8 | 7.5 ± 0.8 | 7.2 ± 0.6 | 6.8 ± 0.4 | 7.2 ± 0.6 | 6.1 ± 1.0 | 5.4 ± 0.6 |
| 1/2 MIC RES | 6.3 ± 0.6^AB^ | 5.7 ± 0.3^B^ | 4.6 ± 0.7^B^ | 6.6 ± 0.5 | 5.5 ± 0.7 | 5.3 ± 0.6 | 7.5 ± 0.7 | 7.3 ± 0.7 | 7.1 ± 0.6 | 7.3 ± 0.6 | 6.5 ± 0.2 | 5.2 ± 0.6 |
| 1/4 MIC RES | 7.3 ± 0.6^A^ | 7.5 ± 0.7^A^ | 4.7 ± 0.8^B^ | 6.5 ± 0.4 | 6.3 ± 0.6 | 5.6 ± 0.6 | 7.5 ± 0.7 | 7.2 ± 0.6 | 6.9 ± 0.6 | 7.3 ± 0.6 | 6.2 ± 0.6 | 6.4 ± 0.6 |
| Control | 6.5 ± 0.4^AB^ | 6.4 ± 0.8^AB^ | 6.6 ± 0.2^A^ | 6.7 ± 0.6 | 6.7 ± 0.5 | 6.1 ± 0.3 | 6.9 ± 0.2 | 7.2 ± 0.4 | 6.7 ± 0.3 | 7.0 ± 0.5 | 6.6 ± 1.0 | 5.5 ± 0.6 |
| Control: 0 µg/mL of QUE or RES.  For *L. plantarum* 49, *L. plantarum* 53 and *L. fermentum* 263 - MIC of QUE: 1024 µg/mL, 1/2 MIC of QUE: 512 µg/mL, 1/4 MIC of QUE: 256 µg/mL, MIC of RES: 1024 µg/mL, 1/2 MIC of RES: 512 µg/mL, 1/4 MIC of RES: 256 µg/mL; For *L. paracasei* 108 and *L. fermentum* 296 - MIC of QUE: 512 µg/mL, 1/2 MIC of QUE: 256 µg/mL, 1/4 MIC of QUE: 128 µg/mL, MIC of RES: 1024 µg/mL, 1/2 MIC of RES: 512 µg/mL, 1/4 MIC of RES: 256 µg/mL; For *L. paracasei* 106 - MIC of QUE: 1024 µg/mL, 1/2 MIC of QUE: 512 µg/mL, 1/4 MIC of QUE: 256 µg/mL, MIC of RES: 1024 µg/mL (assumed), 1/2 MIC of RES: 512 µg/mL (assumed), 1/4 MIC of RES: 256 µg/mL (assumed).  ^A – B^: Different superscript capital letters in the same column, for the same *Lactobacillus* strain, denote difference (p ≤ 0.05) among the counts when exposed when exposed to a bile salts concentration for a specific time interval in media with or without different concentrations of QUE or RES, based on Tukey’s test. The absence of superscript capital letters denotes no difference (p > 0.05) among these counts. | | | | | | | | | | | | |

| **Table S3.** Antagonistic activities, as measured by agar spot test, of different potentially probiotic *Lactobacillus* strains against *Listeria monocytogenes* INCQS 00266 and *Escherichia coli* INCQS 00219 indicator bacteria in the presence, or absence of quercetin (QUE) or resveratrol (RES). The results are expressed as the diameter (mm) of growth inhibition zones (n = 9; average ± standard deviation). | | | | | | | | | | | | | | |
| --- | --- | --- | --- | --- | --- | --- | --- | --- | --- | --- | --- | --- | --- | --- |
| Strains | *L. monocytogenes* INCQS 00266 | | | | | |  | *E. coli* INCQS 00219 | | | | | | |
|  | Control | MIC QUE | 1/2 MIC  QUE | 1/4 MIC QUE | MIC RES | 1/2 MIC RES | MIC/4  RES | Control | MIC QUE | 1/2 MIC QUE | MIC/4 QUE | MIC RES | MIC/2  RES | MIC/4  RES |
| *L. plantarum* 49 | 18.5±2.1 | 16.0±2.8 | 16.0±2.8 | 18.0±2.8 | 20.0±2.8 | 17.5±2.1 | 18.0±2.8 | 16.0±2.8 | 16.0±1.4 | 16.5±2.1 | 20.0±2.8 | 14.5±3.5 | 17.5±2.1 | 17.5±3.5 |
| *L. plantarum* 53 | 14.5±2.1 | 23.0±2.8* | 18.5±1.5* | 17.5±2.1 | 18.0±2.8 | 18.5±3.5 | 16.5±2.1 | 14.5±0.7 | 18.0±1.4* | 17.5±2.1 | 21.0±1.4* | 14.5±0.7 | 15.5±0.7 | 17.0±2.8 |
| *L. paracasei* 106 | 15.5±2.1 | 17.5±2.1 | 19.5±0.7* | 18.5±0.7 | 14.0±1.4 | 17.0±1.4 | 16.5±2.1 | 17.5±3.5 | 14.5±2.1 | 16.5±0.7 | 17.0±2.8 | 16.5±2.1 | 15.5±2.1 | 18.0±2.8 |
| *L. paracasei* 108 | 16.0±1.4 | 19.0±1.4 | 18.0±2.8 | 16.5±2.1 | 19.0±1.4 | 17.5±2.1 | 18.0±2.8 | 14.5±0.7 | 18.5±0.7* | 17.5±1.5* | 18.0±2.8 | 16.5±1.7 | 15.5±0.7 | 12.0±1.4 |
| *L. fermentum* 263 | 19.0±1.4 | 15.5±1.1 | 15.5±2.1 | 17.5±0.8 | 18.0±2.8 | 18.5±0.7 | 18.5±0.7 | 16.5±3.5 | 16.5±0.7 | 16.5±2.1 | 15.5±3.5 | 17.0±1.4 | 18.0±0.7 | 17.5±3.5 |
| *L. fermentum* 296 | 11.0±1.4 | 16.5±0.7* | 21.5±2.1* | 21.5±0.7* | 14.0±1.4 | 16.5±2.1* | 16.0±1.8* | 16.5±2.1 | 17.5±0.8 | 18.0±1.4 | 14.0±2.8 | 15.0±1.4 | 13.5±0.9 | 16.5±2.1 |
| Control: 0 µg/mL of QUE or RES.  For *L. plantarum* 49, *L. plantarum* 53 and *L. fermentum* 263 - MIC of QUE: 1024 µg/mL, 1/2 MIC of QUE: 512 µg/mL, 1/4 MIC of QUE: 256 µg/mL, MIC of RES: 1024 µg/mL, 1/2 MIC of RES: 512 µg/mL, 1/4 MIC of RES: 256 µg/mL; For *L. paracasei* 108 and *L. fermentum* 296 - MIC of QUE: 512 µg/mL, 1/2 MIC of QUE: 256 µg/mL, 1/4 MIC of QUE: 128 µg/mL, MIC of RES: 1024 µg/mL, 1/2 MIC of RES: 512 µg/mL, 1/4 MIC of RES: 256 µg/mL; For *L. paracasei* 106 - MIC of QUE: 1024 µg/mL, 1/2 MIC of QUE: 512 µg/mL, 1/4 MIC of QUE: 256 µg/mL, MIC of RES: 1024 µg/mL (assumed), 1/2 MIC of RES: 512 µg/mL (assumed), 1/4 MIC of RES: 256 µg/mL (assumed).  (*): means difference (p ≤ 0.05) of the measured inhibition zone caused by the tested *Lactobacillus* strain in the presence of QUE or RES in comparison to the control, based on Tukey’s test. | | | | | | | | | | | | | | |
